# Antimicrobial Resistance and Genomic Characteristics of ESBL-Producing *Proteus mirabilis* from Farmed Mink in Shandong Province, China

**DOI:** 10.3390/antibiotics15090933

**Published:** 2026-09-20

**Authors:** Shihao Xuan, Jiayi Sheng, Zunfeng Chu, Min Wang, Huiyang Liu, Yuming Tian, Huajie Zhang, Xiaomin Guo, Jianzhu Liu, Guisheng Wang, Yanhan Liu

**Affiliations:** 1 College of Veterinary Medicine, Shandong Agricultural University, Taian 271018, Chinaliujz@sdau.edu.cn (J.L.); 2 Shandong Provincial Center for Animal Disease Control, Jinan 250100, China; 3 College of Animal Science and Medicine, Shenyang Agricultural University, Shenyang 110161, China; 4 Weihai Animal Epidemic Disease Prevention and Control Center, Weihai 264200, China

**Keywords:** *Proteus mirabilis*, farmed mink, extended-spectrum β-lactamases, antimicrobial resistance, whole-genome sequencing

## Abstract

**Background/Objectives: ***Proteus mirabilis* is an opportunistic pathogen and a potential reservoir of clinically important antimicrobial resistance genes in animal production. However, mink-derived *P. mirabilis* remains poorly characterized. This study investigated multidrug-resistant and extended-spectrum β-lactamase (ESBL)-producing isolates from intensive mink farms, focusing on clinically important β-lactamase genes, their genomic backgrounds, and mobile genetic elements (MGEs). **Methods: ** Among 373 fecal and intestinal-content samples collected from six mink farms in Shandong Province, China, 68 *P. mirabilis* isolates were recovered. All underwent antimicrobial susceptibility testing, ESBL confirmation, and virulence-associated phenotypic assays. The 39 ESBL-positive isolates underwent whole-genome sequencing, followed by analyses of antimicrobial resistance genes, the pangenome, and MGEs and comparison with 198 publicly available genomes. **Results: ** Of the 373 sampled mink, 68 (18.2%) were *P. mirabilis* positive, and 39 (10.5%) carried ESBL-producing isolates. Of the 68 isolates, 63 (92.6%) were multidrug resistant, and 39 (57.4%) were ESBL positive. All exhibited strong biofilm formation and urease activity, while 83.8% showed strong swarming motility. Among the sequenced isolates, *bla*_CTX-M_ genes were identified in 32 isolates and *bla*_NDM-1_ in three. Both *bla*_CTX-M-65_- and *bla*_NDM-1_-positive isolates were distributed across multiple genomic lineages, with *bla*_CTX-M-65_ additionally detected in multiple sequence types. This distribution indicates that these genes were not restricted to a single clone but does not demonstrate horizontal gene transfer. MGE-associated features showed same-contig co-occurrence with several resistance genes, notably *IS903*–*bla*_CTX-M-65_ and *ISAba125*–*bla*_NDM-1_. The sequenced isolates also exhibited substantial accessory-genome diversity and widely distributed virulence determinants associated with adhesion, motility, iron acquisition, and toxin-related functions. **Conclusions: ** Mink-derived *P. mirabilis* exhibited extensive multidrug resistance and carried clinically important β-lactamase genes across diverse genomic backgrounds. These findings support the inclusion of fur-animal production systems in One Health surveillance of antimicrobial-resistant *P. mirabilis*.

## 1. Introduction

*Proteus mirabilis* is a Gram-negative opportunistic pathogen widely distributed in the intestinal tracts of humans and animals and in natural environments. It may persist as an intestinal colonizer but can cause opportunistic infections when host immunity is impaired or epithelial barriers are disrupted [1]. The species possesses multiple virulence-associated traits that facilitate colonization and persistence [2]. Whole-genome studies have further demonstrated substantial genomic diversity in *P. mirabilis* and revealed its capacity to harbor diverse antimicrobial resistance genes (ARGs), virulence-associated genes (VAGs), and mobile genetic elements (MGEs), highlighting its relevance to antimicrobial resistance surveillance in animal-associated bacterial populations [3].

Antimicrobial resistance is a central concern in assessing the risks associated with *P. mirabilis*. Extended-spectrum β-lactamases (ESBLs) represent an important acquired resistance mechanism among Enterobacterales and can reduce susceptibility to penicillins, third-generation cephalosporins, and aztreonam [4]. Among these enzymes, CTX-M-type ESBLs have become globally disseminated β-lactamases of major public health concern [4]. β-Lactamase genes frequently coexist with other ARGs, contributing to multidrug-resistant phenotypes [5]. MGEs, including plasmids, integrons, transposons, and integrative and conjugative elements (ICEs), can facilitate the capture, rearrangement, and dissemination of ARGs [6,7]. Therefore, integrating phenotypic antimicrobial susceptibility testing with whole-genome analysis is important for resolving the genetic basis, genomic context, and potential dissemination of antimicrobial resistance in animal-associated *P. mirabilis*.

Farmed mink represent a distinct but poorly investigated setting in antimicrobial resistance surveillance. Intensive cage-based production concentrates animals, feces, feed, manure, farm personnel, and the surrounding environment within a shared interface, which may facilitate the persistence and circulation of antimicrobial-resistant bacteria and ARGs [8,9,10]. *P. mirabilis* is particularly relevant in this setting because it can persist in both intestinal and environmental reservoirs while carrying diverse ARGs and MGEs [1,3,6,7]. Nevertheless, evidence from mink production remains fragmentary. ESBL-producing bacteria have been recovered from mink feces and feed, with *Proteus* spp. among those detected in fecal samples [11]. A previous study of 53 *P. mirabilis* isolates collectively obtained from fox, raccoon dog, and mink farms in China reported a 73.6% MDR rate and detected clinically important carbapenemase genes, including *bla*_NDM_ and *bla*_OXA-24_ [12]. Similar resistance profiles and close phylogenetic relationships were also observed between isolates from fur animals and the farm environment [12]. However, these studies did not provide a mink-specific characterization of *P. mirabilis.* Consequently, the occurrence and acquired antimicrobial resistance burden of *P. mirabilis* among sampled mink, ESBL production, and the genomic diversity and MGE-associated contexts of the ESBL-positive subset remain insufficiently characterized.

To address these knowledge gaps, we investigated *P. mirabilis* recovered from intensive mink farms in Shandong Province, China. Antimicrobial resistance and virulence-associated phenotypes were characterized for all isolates, followed by whole-genome sequencing of the ESBL-positive subset. Particular emphasis was placed on clinically important β-lactamase genes, their distribution across genomic backgrounds, and their genomic co-occurrence with MGE-associated regions. Virulence-associated genes and pangenome diversity were further characterized, and publicly available genomes were included to place the mink-derived isolates within a broader population genomic context.

## 2. Materials and Methods

### 2.1. Sample Collection and Bacterial Identification

From 5 June 2025 to 18 March 2026, 373 samples were collected from six large-scale commercial mink farms in Weihai, Weifang, Linyi, and Yantai, Shandong Province, China. The farms were located in areas where commercial mink production was relatively concentrated and were included if they used standardized cage-based housing, were in active production during the sampling period, agreed to participate, and permitted sample collection. The samples comprised 270 freshly voided fecal samples collected non-invasively from randomly selected individual cages and 103 postmortem intestinal-content samples. Fecal samples were collected irrespective of the apparent clinical status of the mink, and carcasses were selected without reference to their premortem health history. At each postmortem sampling event, one of the first ten eligible carcasses was randomly selected, followed by sampling of every tenth carcass until the planned number was reached. Selection of cages and carcasses followed the general sampling principles of NY/T 4139-2022 [13]. Postmortem samples were obtained from carcasses following routine commercial pelting performed independently by farm personnel; no animals were killed specifically for this study. One sample was collected per animal, and one confirmed *Proteus mirabilis* isolate per positive sample was retained. Samples were aseptically collected into sterile tubes and transported to the laboratory under cold-chain conditions on the day of collection. All sampling procedures were approved by the Institutional Animal Care and Use Committee of Shandong Agricultural University (approval No. SDAUA-2025-173).

Samples were inoculated into Luria–Bertani broth (LB; Qingdao Hope Bio-Technology Co., Ltd., Qingdao, China) and incubated at 37 °C with agitation at 220 rpm for 16 h. After enrichment, the cultures were streaked onto LB agar plates and incubated at 37 °C for 16 h. Colonies exhibiting the characteristic swarming phenotype were presumptively identified as *P. mirabilis*. Presumptive colonies were subsequently subcultured on Salmonella–Shigella agar (SS agar; Qingdao Hope Bio-Technology Co., Ltd., Qingdao, China) and incubated at 37 °C for 16 h. Colorless or translucent colonies with black centers were selected for further identification.

Purified presumptive isolates were initially identified using matrix-assisted laser desorption/ionization time-of-flight mass spectrometry (MALDI-TOF MS; Clin-TOF II, Bioyong Technologies Inc., Beijing, China). Identification results were interpreted according to the criteria recommended by the instrument manufacturer, and all isolates included in subsequent analyses had confidence values greater than 70. MALDI-TOF MS identifications were subsequently confirmed using a PCR assay targeting the *P. mirabilis*-specific *ureR* gene. The primers used were ureRF1 (5′-GGTGAGATTTGTATTAATGG-3′) and ureRR1 (5′-ATAATCTGGAAGATGACGAG-3′), which were expected to amplify a 225-bp fragment. PCR was performed in a final reaction volume of 25 μL under the following cycling conditions: initial denaturation at 94 °C for 4 min; 30 cycles of denaturation at 94 °C for 40 s, annealing at 58 °C for 1 min, and extension at 72 °C for 20 s; followed by a final extension at 72 °C for 10 min. Isolates yielding concordant identification results by MALDI-TOF MS and *ureR*-specific PCR as *P. mirabilis* were further analyzed.

### 2.2. Antimicrobial Susceptibility Testing

Antimicrobial susceptibility testing was performed using the broth microdilution method in accordance with the Clinical and Laboratory Standards Institute (CLSI) guidelines [14]. Analytical standards of the antimicrobial agents were obtained from Shanghai Macklin Biochemical Co., Ltd. (Shanghai, China) and serially twofold diluted in cation-adjusted Mueller–Hinton broth (CAMHB; Qingdao Hope Bio-Technology Co., Ltd., Qingdao, China) in 96-well microtiter plates. Bacterial suspensions were prepared from purified cultures and adjusted to yield a final inoculum of approximately 5 × 10^5^ CFU/mL in each well, with a final volume of 100 μL per well. The inoculated plates were incubated at 37 °C for 18–24 h. The minimum inhibitory concentration (MIC) was defined as the lowest concentration of an antimicrobial agent that completely inhibited visible bacterial growth.

A total of 11 antimicrobial agents were tested: doxycycline (DO), imipenem (IPM), cefepime (FEP), gentamicin (GEN), amikacin (AMK), enrofloxacin (ENR), cefoxitin (FOX), ampicillin (AM), florfenicol (FFC), trimethoprim–sulfamethoxazole (SXT), and aztreonam (ATM). MICs for DO, IPM, FEP, GEN, AMK, FOX, AM, SXT, and ATM were interpreted using the Enterobacteriaceae criteria presented in CLSI VET08, 4th ed. (2018), Table S4, and adopted from CLSI M100, 28th ed. (2018) [14,15]. In the absence of mink-specific breakpoints, the FDA-approved canine/feline criterion was used as a surrogate for phenotypic categorization of ENR [16], whereas the swine-derived florfenicol criterion for Enterobacterales specified in CLSI VET01S, 7th ed. (2024), was used as a surrogate for epidemiological interpretation of FFC [17]. The complete interpretive criteria and antimicrobial-category assignments are provided in Table S4. Following a previously proposed framework [18], MDR was defined as acquired resistance to at least one tested agent in three or more antimicrobial categories. ENR and FFC were included as fluoroquinolone and phenicol agents, respectively, using the surrogate interpretive criteria described above, whereas DO was excluded because *P. mirabilis* is intrinsically resistant to tetracyclines [19]. Each isolate–antimicrobial combination was tested in triplicate, and the median MIC was used for final susceptibility categorization. *Escherichia coli* ATCC 25922 was used as the quality-control strain.

ESBL production was phenotypically assessed using the CLSI-recommended combination disk method [14]. Disks containing cefotaxime, cefotaxime/clavulanic acid, ceftazidime, or ceftazidime/clavulanic acid were obtained from BD (USA). An isolate was classified as phenotypically ESBL positive when the inhibition-zone diameter produced by either cefotaxime/clavulanic acid or ceftazidime/clavulanic acid was at least 5 mm greater than that produced by the corresponding cephalosporin disk alone.

### 2.3. Assessment of Biofilm-Forming Capacity

Biofilm formation was assessed using a crystal violet staining assay in 96-well polystyrene microtiter plates. Following overnight culture in LB broth, the bacterial suspensions were adjusted to an optical density at 600 nm (OD_600_) of 0.1. Aliquots of 200 μL were transferred to individual wells, while wells containing only LB broth served as negative controls. Each isolate was tested in three replicate wells in three independent experiments. After incubation at 37 °C for 24 h, the culture medium was removed, and the wells were gently washed with sterile phosphate-buffered saline (PBS) to remove non-adherent cells. The attached cells were fixed with 200 μL of methanol for 15 min, after which the methanol was removed and the plates were air-dried. Each well was stained with 1% crystal violet for 30 min. Excess stain was removed by washing with sterile water, and the plates were air-dried. The bound crystal violet was subsequently solubilized with 33% acetic acid for 30 min at room temperature, and absorbance was measured at 570 nm. For each isolate, the mean absorbance of the three replicate wells was calculated for each independent experiment, and the mean of the three experiment-level values was used for statistical analysis. The optical density cutoff (OD_c_) was defined as the mean OD_570_ of the negative controls plus three standard deviations. Biofilm-forming capacity was classified as follows: OD_570_ ≤ OD_c_, non-biofilm producer; OD_c_ < OD_570_ ≤ 2 × OD_c_, weak biofilm producer; 2 × OD_c_ < OD_570_ ≤ 4 × OD_c_, moderate biofilm producer; and OD_570_ > 4 × OD_c_, strong biofilm producer [20].

### 2.4. Swarming Motility Assay

The test isolates were cultured to the logarithmic growth phase, and the bacterial suspensions were adjusted to an optical density at 600 nm (OD_600_) of 0.4. A 5 μL aliquot of each suspension was spotted onto the center of an LB agar plate containing 1.5% agar. After the inoculum had been completely absorbed, the plates were inverted and incubated at 37 °C for 9 h. The maximum diameter of the swarming zone was measured after incubation, and the expansion rate was calculated by dividing this diameter by the 9-h incubation period. Swarming motility was classified according to the proportion of the agar surface covered by bacterial growth: coverage ≤ 5%, weak swarming; 5% < coverage ≤ 25%, moderate swarming; and coverage > 25%, strong swarming [21]. Each isolate was tested in three independent experiments, and the mean of the three swarming-zone diameter measurements was used for classification and statistical analysis.

### 2.5. Hemolytic and Urease Activities

Hemolytic activity was assessed using the blood agar plate method. Purified isolates were inoculated onto blood agar plates (catalog no. 024070; Guangdong Huankai Microbial Sci. & Tech. Co., Ltd., Guangzhou, China) containing 5% defibrinated sheep blood and incubated at 37 °C for 24 h. Following incubation, the plates were examined for clear zones of hemolysis surrounding the colonies. The presence of a clear zone was recorded as β-hemolytic activity.

Urease activity was assessed using urease test medium in biochemical tubes. A single purified colony was inoculated into the medium and incubated at 37 °C for 24 h. A color change from orange-yellow to rose-red was interpreted as a positive urease reaction, whereas the absence of an appreciable color change was interpreted as a negative reaction.

### 2.6. Whole-Genome Sequencing and Bioinformatic Analyses

All 39 isolates with a confirmed ESBL-positive phenotype were subjected to whole-genome sequencing (WGS). Genomic DNA was extracted using a Roche MagNA Pure 24 automated nucleic acid extraction system and the corresponding extraction kit. DNA integrity, concentration, and purity were assessed, and samples meeting the quality requirements were used for library preparation. Whole-genome sequencing was performed by Sangon Biotech (Shanghai) Co., Ltd. (Shanghai, China) using a DNBSEQ-T7 platform (BGI Group, Shenzhen, China). Raw reads were processed using Trimmomatic v0.36 to remove adapter sequences and low-quality reads. The resulting clean reads were assembled using SPAdes v3.5.0, and remaining gaps were filled using GapFiller v1.11. Taxonomic identification of the 39 genome assemblies was confirmed by ANI analysis using skani v0.3.2 in slow mode against the *P. mirabilis* type strain ATCC 29906 (GCF_000160755.1), with ≥95% ANI used as the species-level threshold [22].

Gene prediction and genome annotation were performed using Prokka v1.14.6 [23]. Potential acquired antimicrobial resistance genes (ARGs) were identified using ABRicate v1.4.0 [24] against the CARD [25] and ResFinder databases, with minimum identity and coverage thresholds of 80%. Protein sequences were aligned against VFDB Set B [26] using DIAMOND v2.0.8 [27] for the identification of virulence-associated genes (VAGs), with an identity threshold of >90% and an E-value cutoff of 1 × 10^−5^. Pangenome analysis was performed using Roary v3.13.0 [28], and a maximum-likelihood phylogenetic tree was constructed from the core-gene alignment using IQ-TREE v3.1.2 [29].

Insertion sequences and transposons were identified using ISFinder [30], integrons using IntegronFinder v2.0.2 [31], integrative and conjugative elements (ICEs) using ICEfinder 2.0 [32], and prophage regions using PHASTEST [33]. Plasmid-associated sequences were extracted from Prokka annotations based on genes associated with plasmid replication, partition, or mobilization [23] and were interpreted as annotation-derived features rather than reconstructed complete plasmids. Annotation-based genomic co-occurrence analysis and subsequent data processing and visualization were performed using Python v3.10.20. Based on genomic coordinates derived from the short-read assemblies, co-occurrence was operationally defined as an ARG or VAG located within a putative MGE-associated region or within 5 kb of its boundary on the same contig.

Phenotypic experiments were performed in triplicate. Differences between two groups were evaluated using the two-sided Mann–Whitney *U* test. Spearman’s rank correlation analysis was used to evaluate associations between variables. For resistance-related correlations, resistant results were coded as 1, whereas susceptible, intermediate, and susceptible-dose-dependent results were coded as 0. The overall phenotypic resistance burden was defined as the number of resistant results among all 11 tested antimicrobial agents, including DO, ENR, and FFC; DO was excluded only from MDR classification. Statistical analyses were performed using Python v3.10.20 and SciPy v1.13.1. No correction for multiple comparisons was applied; all *p*-values were two-sided and unadjusted, with *p* < 0.05 used as an exploratory threshold.

### 2.7. Public Genome Comparison and Population Structure Analysis

To place the mink-derived *Proteus mirabilis* isolates within a broader population genomic context, 198 publicly available *P. mirabilis* genomes representing different host sources were downloaded from the NCBI GenBank database and analyzed together with the 39 mink-derived isolates sequenced in this study. Host information for the public genomes was retrieved from the corresponding NCBI metadata records and classified as human/clinical, swine, poultry, companion animal, livestock, other animal, or unknown. The GenBank accession numbers and core metadata for the 198 public genomes are provided in Supplementary Table S2. The public genomes were included solely to provide a broader genomic background and were excluded from analyses of phenotypic antimicrobial resistance.

Sequence types (STs) were assigned using mlst v2.33.1 and the six-locus *Proteus* spp. MLST scheme (*atpD*, *dnaJ*, *mdh*, *pyrC*, *recA*, and *rpoD*) available in the PubMLST database [34,35]. Antimicrobial resistance genes were identified using ABRicate v1.4.0 against the ResFinder and CARD databases and subsequently grouped according to major antimicrobial classes. Pairwise genomic distances were calculated from the assembled genomes using Mash v2.3 [36], and a distance-based clustering tree was generated from the resulting distance matrix. The host sources, major STs, and selected antimicrobial resistance gene classes were visualized using the Interactive Tree of Life (iTOL).

## 3. Results

### 3.1. Isolation and Identification of Proteus mirabilis

**Figure 1:**
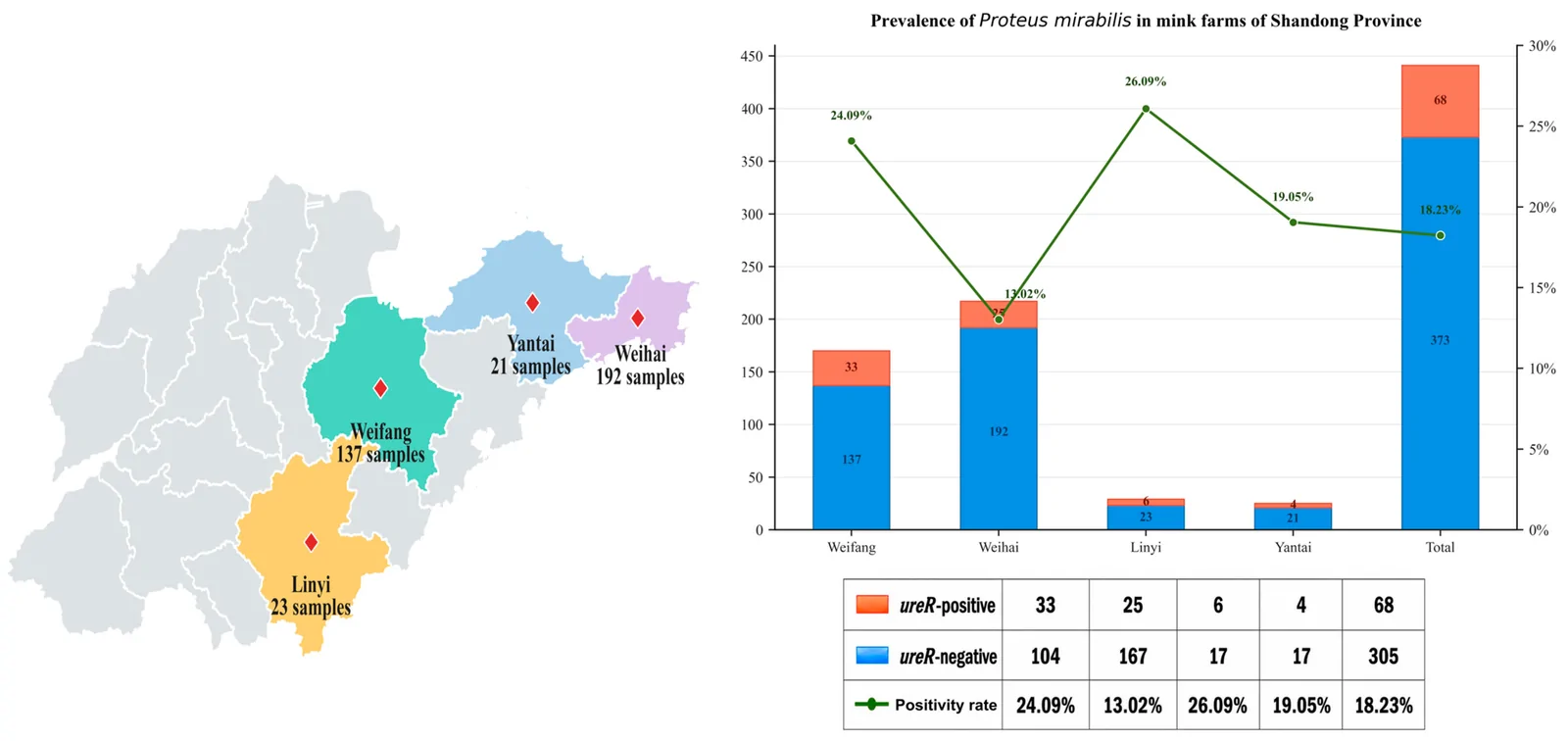
Isolation rates of *Proteus mirabilis* from mink farms in Shandong Province, China.

A total of 373 fecal and intestinal-content samples were collected from six large-scale mink farms in Shandong Province, with one sample obtained from each animal. Following selective culture, preliminary identification by MALDI-TOF MS, and confirmation by *ureR*-specific PCR, *P. mirabilis* was recovered from 68 of the 373 sampled mink, corresponding to an overall animal-level positivity rate of 18.2% (68/373). The isolation rates varied among sampling locations (Figure 1), reaching 24.1% (33/137) in Weifang, 13.0% (25/192) in Weihai, 26.1% (6/23) in Linyi, and 19.0% (4/21) in Yantai.

### 3.2. Antimicrobial Susceptibility and ESBL Phenotypes of Proteus mirabilis Isolates

**Figure 2:**
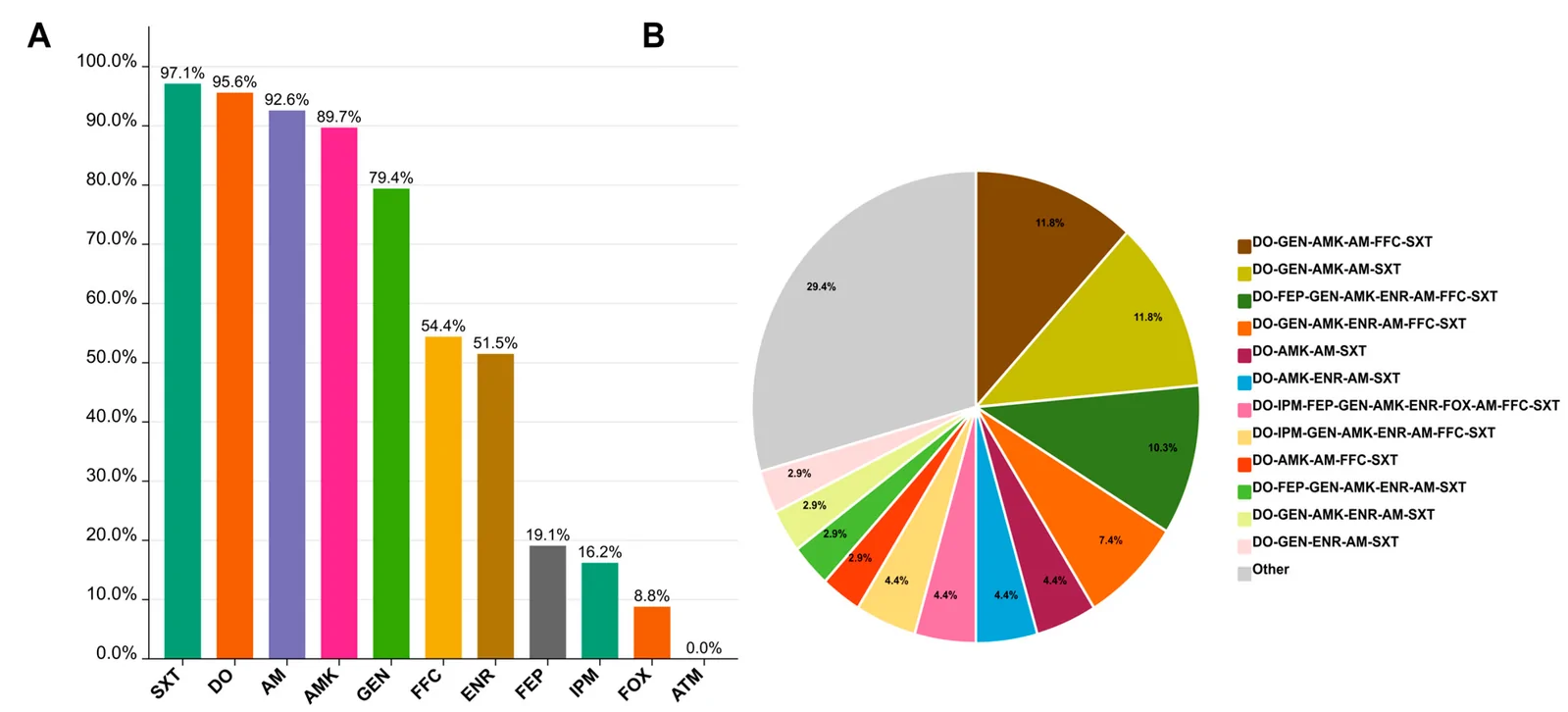
Phenotypic antimicrobial resistance and resistance-profile distribution of 68 mink-derived *Proteus mirabilis* isolates. (**A**) Resistance rates of the isolates to 11 antimicrobial agents. (**B**) Frequencies of the 30 phenotypic resistance profiles identified among the isolates.

At the isolate level, the MIC ranges, MIC_50_ and MIC_90_ values, and resistance rates of the 68 *P. mirabilis* isolates against 11 antimicrobial agents are summarized in Table 1. Phenotypic resistance rates and the distribution of resistance profiles are shown in Figure 2A,B. The highest resistance rate was observed for trimethoprim–sulfamethoxazole (SXT; 97.1%, 66/68), followed by doxycycline (DO; 95.6%, 65/68), ampicillin (AM; 92.6%, 63/68), amikacin (AMK; 89.7%, 61/68), and gentamicin (GEN; 79.4%, 54/68). Resistance to florfenicol (FFC) and enrofloxacin (ENR) was detected in 54.4% (37/68) and 51.5% (35/68) of the isolates, respectively. Lower resistance rates were observed for cefepime (FEP; 19.1%, 13/68), imipenem (IPM; 16.2%, 11/68), and cefoxitin (FOX; 8.8%, 6/68). None of the isolates was classified as resistant to aztreonam (ATM).

The 68 isolates exhibited 30 distinct phenotypic resistance profiles (Figure 2B; Supplementary Table S1), indicating substantial diversity in their resistance phenotypes. DO–GEN–AMK–AM–FFC–SXT and DO–GEN–AMK–AM–SXT were the two most common profiles, each detected in eight isolates (11.8%, 8/68). These were followed by DO–FEP–GEN–AMK–ENR–AM–FFC–SXT, detected in seven isolates (10.3%, 7/68), and DO–GEN–AMK–ENR–AM–FFC–SXT, detected in five isolates (7.4%, 5/68). Each of the remaining resistance profiles was detected in one to three isolates. After exclusion of doxycycline from the MDR calculation, 63 of the 68 isolates (92.6%) met the predefined MDR criterion.

Phenotypic confirmatory testing identified 39 ESBL-positive isolates (57.4%, 39/68), corresponding to 10.5% (39/373) of all samples. All ESBL-positive isolates were subsequently included in the whole-genome sequencing analysis. These findings indicate that MDR was widespread among the mink-derived *P. mirabilis* isolates and that ESBL-positive isolates constituted a substantial proportion of the collection.

### 3.3. Virulence-Associated Phenotypes of Proteus mirabilis Isolates

All 68 *P. mirabilis* isolates formed biofilms. Based on the OD_c_ classification criteria, the OD_c_ and 4 × OD_c_ values were 0.231 and 0.924, respectively, and the mean OD_570_ value for every isolate exceeded 4 × OD_c_. PM-C106 exhibited the greatest biofilm-forming capacity, with a mean OD_570_ value of 3.27, whereas PM-C38 had the lowest mean OD_570_ value (1.25) but was still classified as a strong biofilm producer. Biofilm-forming capacity did not differ significantly between ESBL-positive and ESBL-negative isolates (*p* > 0.05).

All 68 isolates exhibited swarming motility to varying degrees. Of these, 57 (83.8%) were classified as having strong swarming motility and 11 (16.2%) as having moderate swarming motility; no isolate exhibited weak swarming motility. PM-C03, PM-C08, and PM-C20 showed the greatest swarming motility, each with an expansion diameter of 83.0 mm and an expansion rate of 9.22 mm/h. PM-A32 showed the lowest swarming motility, with an expansion diameter of 23.3 mm and an expansion rate of 2.59 mm/h. No significant difference in swarming motility was observed between the ESBL-positive and ESBL-negative isolates (*p* > 0.05).

None of the 68 isolates produced a characteristic clear β-hemolytic zone on 5% sheep blood agar, indicating no detectable β-hemolytic activity under the conditions tested. In the urease assay, all isolates changed the medium from orange-yellow to rose-red, and they were therefore classified as urease positive.

### 3.4. Pangenome Structure and Accessory-Genome Diversity of ESBL-Positive Isolates

**Figure 3:**
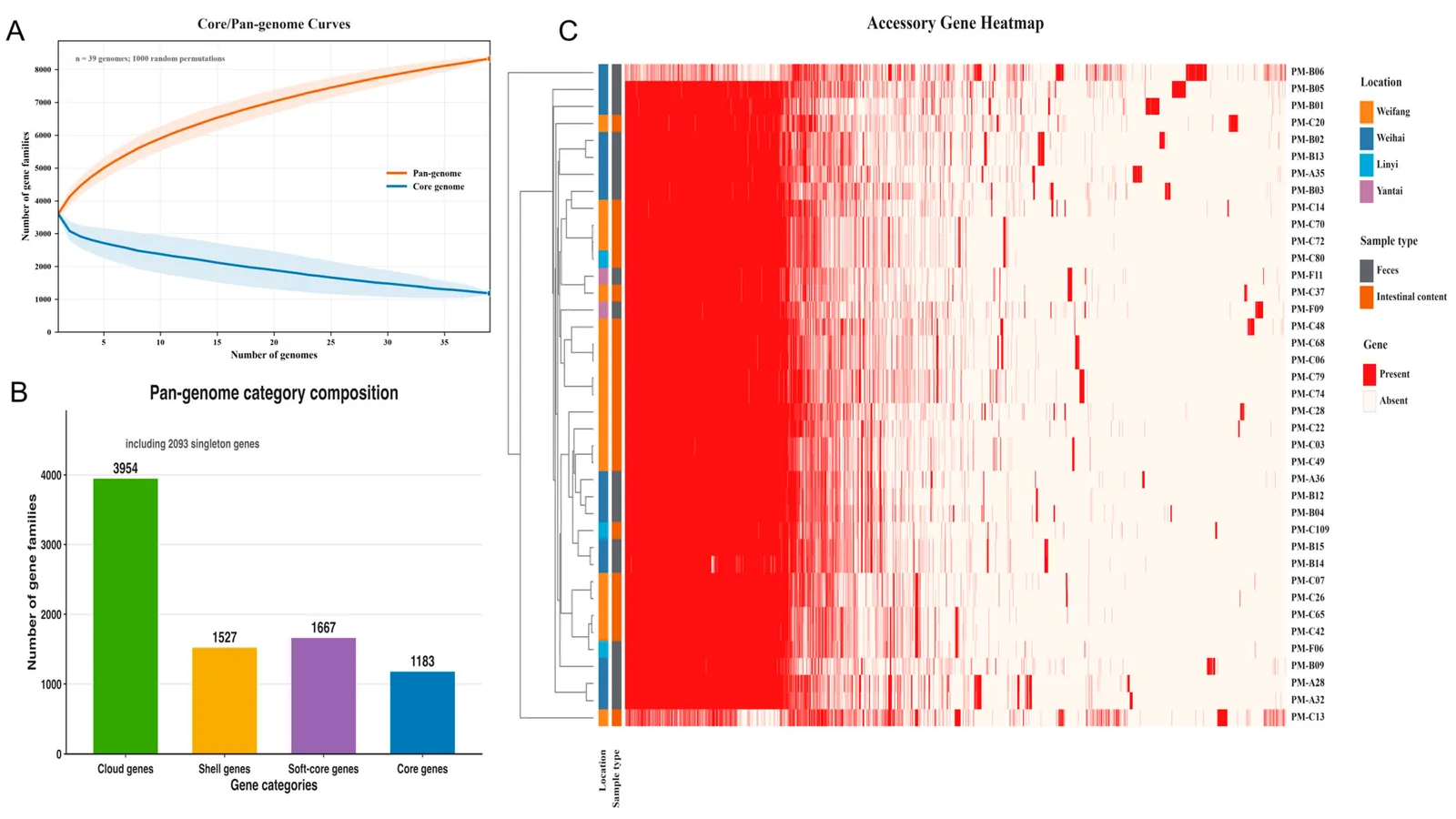
Pangenome structure and accessory gene profiles of the *Proteus mirabilis* isolates. (**A**) Core- and pan-genome curves generated from the gene presence–absence matrix of the 39 ESBL-positive *P. mirabilis* isolates. The orange curve represents the pangenome, and the blue curve represents the core genome. (**B**) Distribution of pangenome categories among the 39 isolates. Gene families were classified as cloud, shell, soft-core, or core genes according to their frequency across the isolates. (**C**) Heatmap showing the presence or absence of accessory genes. Red indicates gene presence, whereas white indicates gene absence. The annotation bars on the left indicate sampling location and sample type.

All 39 sequenced isolates were confirmed as *P. mirabilis* by ANI analysis, with values ranging from 98.44% to 99.25% relative to the type strain ATCC 29906 (Table S5). Subsequent pangenome analysis of the gene presence–absence matrix for the 39 ESBL-positive *P. mirabilis* isolates identified 8331 gene families (Figure 3A). Of these, 1183 were present in all isolates and constituted the core genome, whereas the remaining 7148 constituted the non-core genome. As the number of genomes included in the analysis increased, the size of the core genome gradually decreased and approached a plateau, whereas the pangenome continued to expand. This pattern indicates substantial variation in gene content and considerable genomic plasticity among the mink-derived isolates.

The gene families were further classified according to their frequency among the 39 isolates. Cloud genes represented the largest category, comprising 3954 gene families, of which 2093 were isolate-specific singleton gene families. The remaining categories comprised 1667 soft-core, 1527 shell, and 1183 core gene families (Figure 3B). The high proportions of cloud and shell gene families indicate that non-conserved genetic components contributed substantially to the genomic variation observed among the isolates.

The accessory gene presence–absence heatmap revealed marked heterogeneity in accessory gene content among the isolates. Although some isolates displayed similar accessory gene profiles, no distinct clustering pattern consistently associated with geographic origin or sample type was observed (Figure 3C). Overall, the mink-derived *P. mirabilis* isolates possessed a diverse accessory gene pool and exhibited substantial accessory-genome diversity.

### 3.5. Antimicrobial Resistance Gene Profiles of ESBL-Positive Isolates

**Figure 4:**
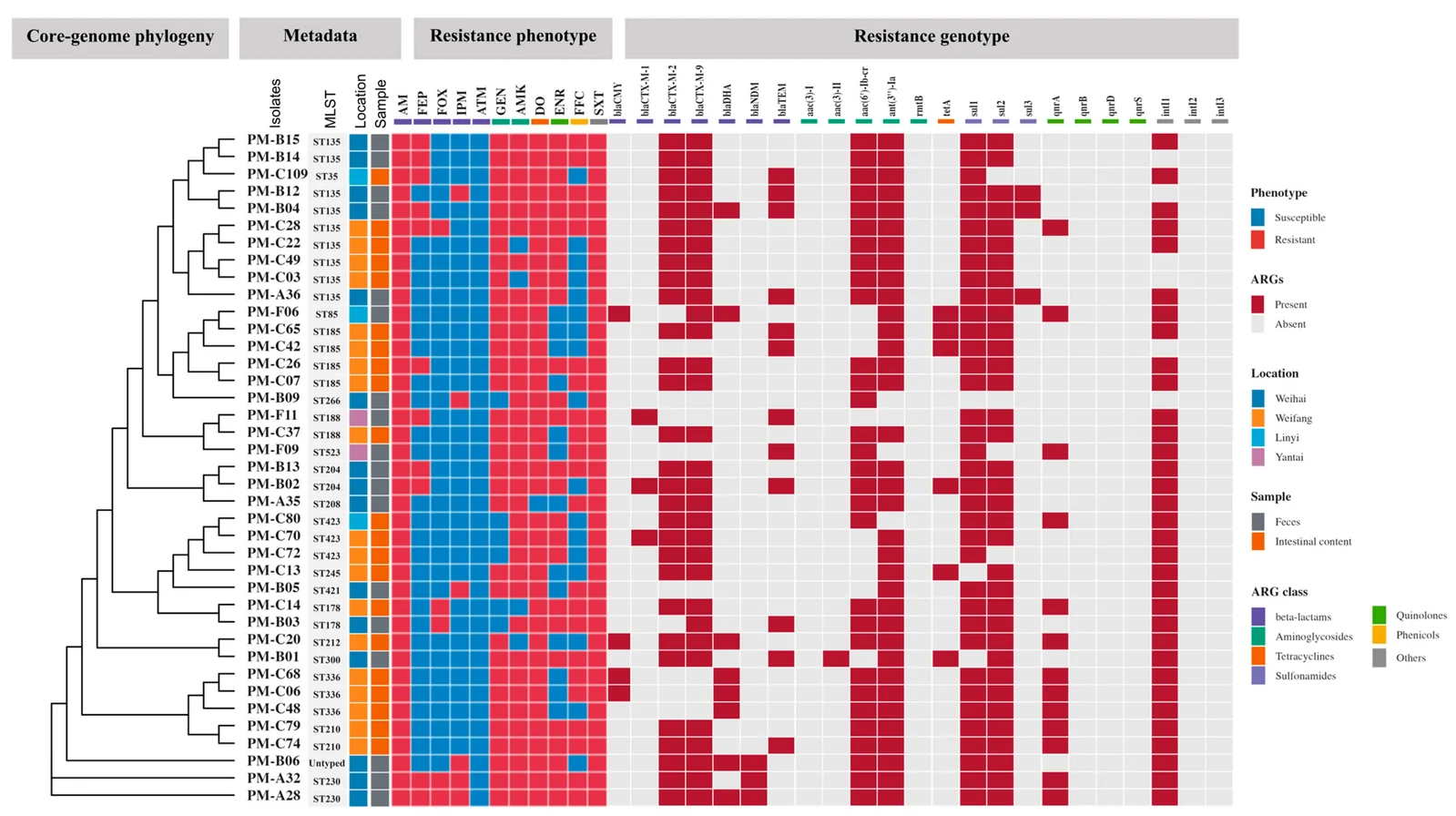
Phylogenetic distribution of antimicrobial resistance phenotypes and resistance genes among *Proteus mirabilis* isolates. The core-genome phylogenetic tree is shown on the left. The adjacent metadata tracks indicate isolate identifier, MLST sequence type (ST), sampling location, and sample type. The antimicrobial resistance phenotype heatmap shows the MIC-based resistance profiles of the 39 sequenced isolates to 11 antimicrobial agents, with blue and red representing non-resistant and resistant phenotypes, respectively. The resistance genotype heatmap shows the presence or absence of antimicrobial resistance genes (ARGs) identified by whole-genome sequence analysis; dark red indicates gene presence, whereas light gray indicates gene absence. The annotation bars at the top indicate the corresponding antimicrobial classes and ARG categories. All metadata tracks and heatmaps are arranged according to the isolate order in the phylogenetic tree.

A core-genome phylogeny of the 39 ESBL-positive *P. mirabilis* isolates was analyzed together with isolate metadata, antimicrobial resistance phenotypes, and ARG profiles (Figure 4). Isolates from different geographic locations and sample types did not form distinct phylogenetic clusters, and no clear correspondence was observed between phylogenetic relatedness and either geographic origin or sample type.

All 39 sequenced isolates exhibited multidrug-resistant phenotypes involving β-lactams, aminoglycosides, sulfonamides, tetracyclines, quinolones, and phenicols. Most isolates were resistant to ampicillin, doxycycline, trimethoprim–sulfamethoxazole, gentamicin, and amikacin, whereas none was classified as resistant to aztreonam. ARG annotation identified multiple resistance gene classes, among which β-lactamase, sulfonamide-resistance, and aminoglycoside-resistance genes were the most frequently detected. Genes of the *bla*_CTX-M_ family were identified in 32 of the 39 ESBL-positive isolates, indicating that CTX-M-type ESBL genes were a major genetic determinant of β-lactam resistance in the mink-derived isolates.

In addition to *bla*_CTX-M_, some isolates carried other β-lactamase genes, including *bla*_TEM_, *bla*_DHA_, and *bla*_CMY_, demonstrating the complexity of their β-lactam resistance backgrounds. Notably, *bla*_NDM-1_ was detected in PM-B06, PM-A28, and PM-A32, revealing the presence of a carbapenemase gene in a subset of the mink-derived isolates and highlighting its potential public health relevance. The *sul1*, *sul2*, *ant(3″)-Ia*, and *aac(6′)-Ib-cr* genes were widely distributed, whereas *qnrA* and *tetA* were the principal quinolone- and tetracycline-resistance genes, respectively. The class 1 integron integrase gene *intI1* was detected in most isolates, suggesting that integrons may contribute to the maintenance or dissemination of some ARGs.

### 3.6. Virulence-Associated Gene Profiles of Proteus mirabilis Isolates

**Figure 5:**
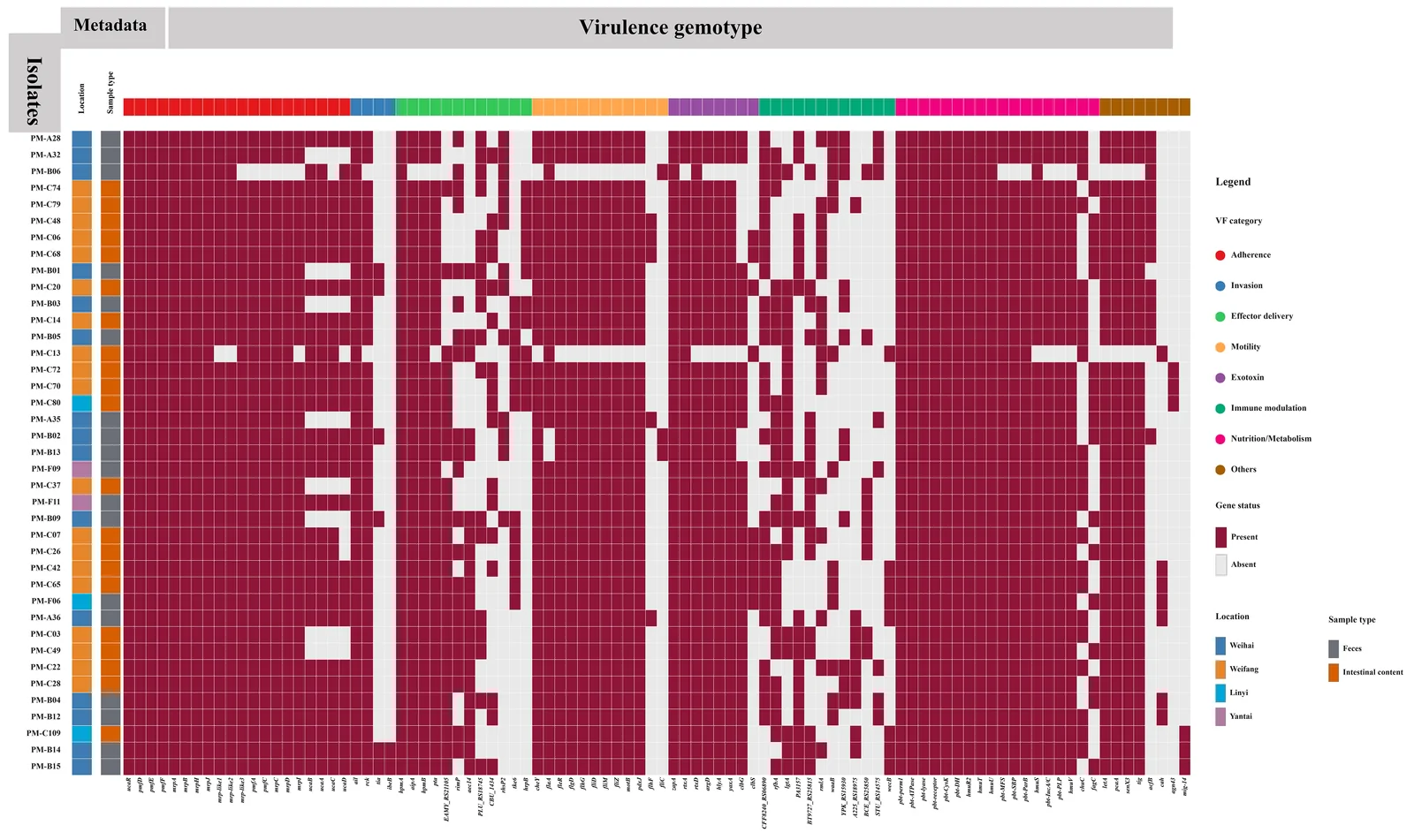
Virulence-associated gene profiles of 39 mink-derived *Proteus mirabilis* isolates. The heatmap shows the presence or absence of 94 representative virulence-associated genes selected from VFDB Set B annotations. The genes were assigned to eight functional categories: adherence, invasion, effector delivery, motility, exotoxin, immune modulation, nutrition/metabolism, and other functions. Annotation bars on the left indicate sampling location and sample type. Red indicates gene presence, whereas light gray indicates gene absence.

Annotation against VFDB Set B yielded 592 virulence-associated gene (VAG) hits across the 39 mink-derived *P. mirabilis* isolates. To reduce redundancy caused by homologous matches, 94 representative VAGs were selected for visualization and analysis (Figure 5). These VAGs were assigned to eight functional categories: adherence, invasion, effector delivery, motility, exotoxins, immune modulation, nutrition/metabolism, and other functions. Individual isolates carried 42–77 representative VAGs, with a mean of 71.1. Twenty VAGs were detected in all isolates, constituting a conserved virulence-associated gene set.

Genes associated with adherence and nutrition/metabolism were particularly abundant. The MR/P fimbrial genes *mrpA*, *mrpB*, *mrpH*, and *mrpJ*, together with the PMF fimbrial genes *pmfD*, *pmfE*, and *pmfF*, were detected in all isolates. Genes associated with the proteobactin and HmuR2 iron-acquisition systems, including *pbt-perm1*, *pbt-ATPase*, *pbt-receptor*, *hmuR2*, *hmuT*, and *hmuU*, were also detected in most isolates. In addition, the exotoxin- and effector delivery-associated genes *zapA*, *hpmA*, *hpmB*, *aipA*, and *pta*, as well as the motility-associated genes *flaA*, *flgD*, *fliD*, *fliM*, *fliZ*, and *motB*, were frequently detected. These findings indicate that the mink-derived isolates commonly carried genetic determinants associated with adherence, iron acquisition, motility, and tissue damage.

Most isolates exhibited broadly similar VAG profiles. However, PM-B06 and PM-C13 carried comparatively fewer representative VAGs, with 42 and 47 genes, respectively, suggesting that their VAG repertoires differed from those of the other isolates. Overall, the mink-derived *P. mirabilis* isolates carried diverse categories of VAGs, indicating a broad repertoire of genetic determinants potentially associated with virulence.

### 3.7. Distribution of MGE-Associated Features and Their Correlations with Resistance and Virulence Traits

**Figure 6:**
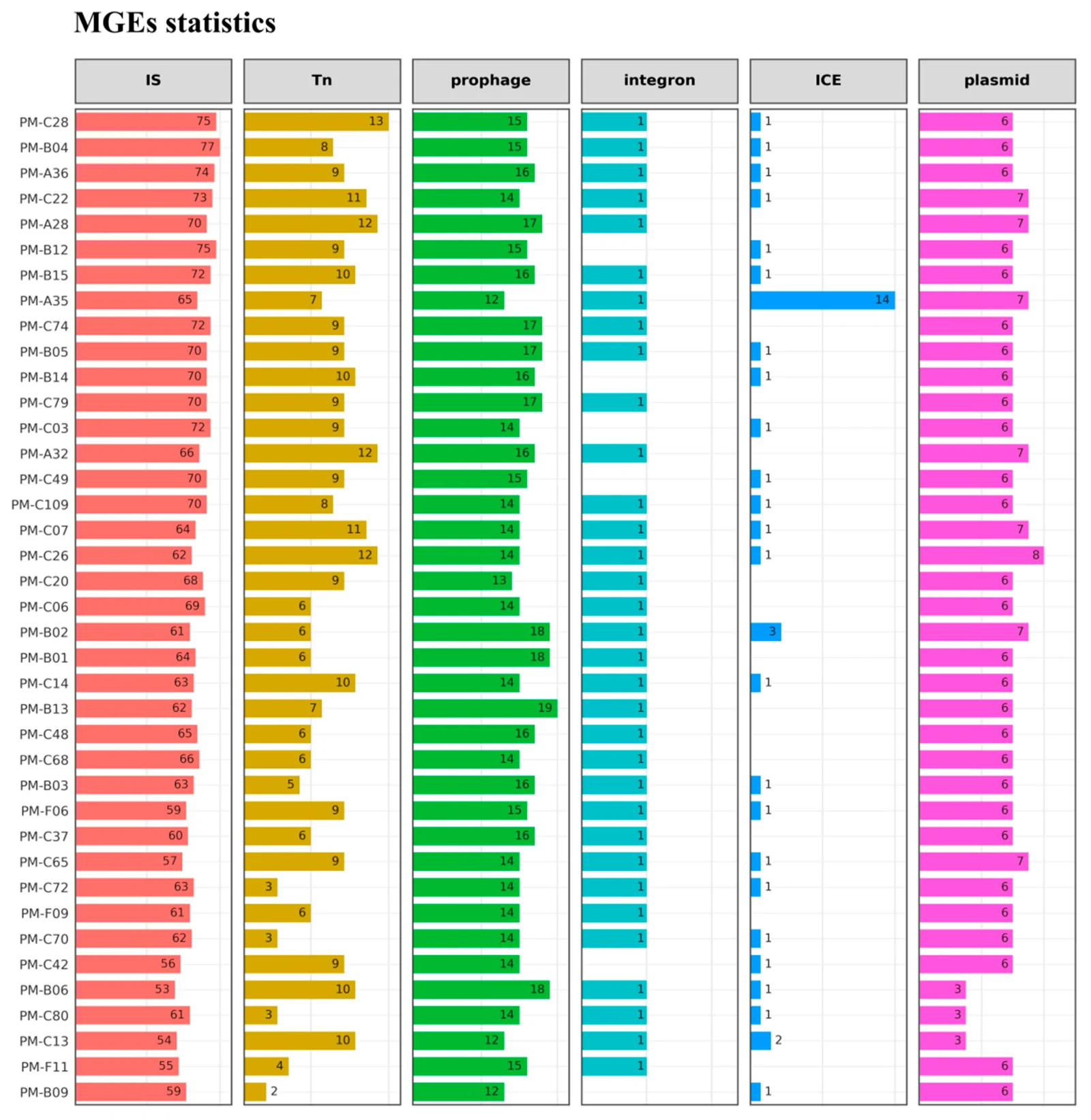
Distribution of MGE-associated features among 39 mink-derived *Proteus mirabilis* isolates. The figure shows counts of six categories of MGE-associated features detected in each isolate: insertion sequences (ISs), transposons (Tns), integrons, prophages, ICE/conjugation-associated elements, and plasmid-associated sequences. Bars indicate the count of each category per isolate and illustrate variation in MGE composition and burden among isolates.

To characterize MGE distribution in the genomes of mink-derived *P. mirabilis*, MGE-associated features were quantified in the 39 sequenced isolates from genome annotation and MGE prediction results. A total of 3757 MGE-associated features were identified. Insertion sequences (ISs) were the most abundant category (*n* = 2548), followed by prophage-associated features (*n* = 588), transposons (Tns; *n* = 312), plasmid-associated sequences (*n* = 234), and integrative and conjugative element (ICE)/conjugation-associated features (*n* = 42). The remaining 33 annotations were integron associated. Individual isolates carried 80–111 MGE-associated features, with a mean of 96.3, indicating that diverse MGE-related sequences were widely distributed among the mink-derived *P. mirabilis* isolates (Figure 6).

**Figure 7:**
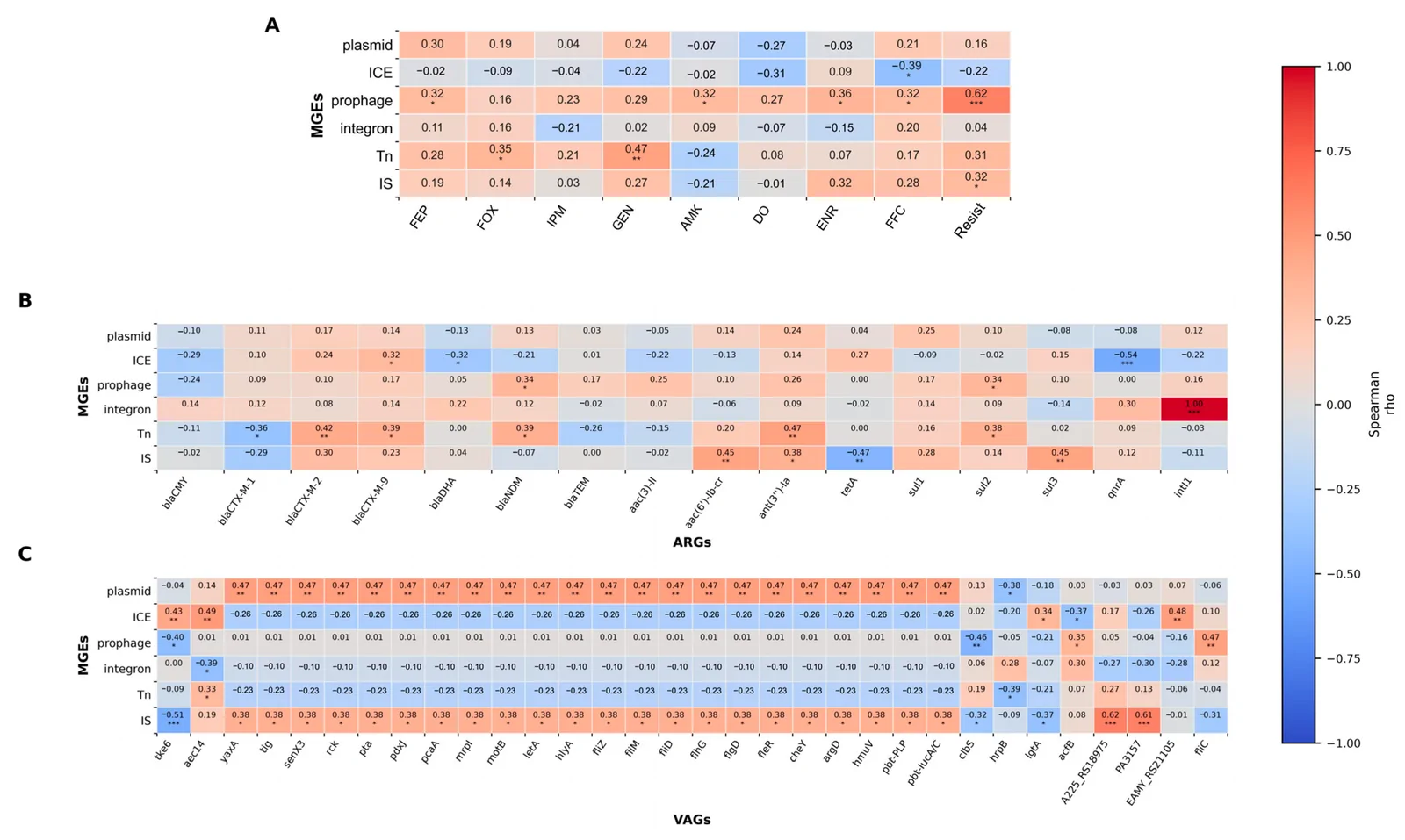
Correlations of MGE-associated features with antimicrobial resistance phenotypes, ARGs, and VAGs. The heatmaps show Spearman correlations between six MGE categories and (**A**) antimicrobial resistance phenotypes, (**B**) ARGs, and (**C**) representative VAGs. Values within cells are Spearman’s *ρ* coefficients; red indicates positive correlations, whereas blue indicates negative correlations. Asterisks indicate unadjusted *p*-value thresholds: * *p* < 0.05, ** *p* < 0.01, and *** *p* < 0.001. The correlations were considered exploratory.

Spearman’s correlation analysis showed that, at the unadjusted exploratory threshold, prophage-associated feature abundance was positively correlated with FEP, AMK, ENR, and FFC resistance and with the overall phenotypic resistance burden (*ρ* = 0.318, 0.321, 0.358, 0.317, and 0.615; *p* = 0.048, 0.046, 0.025, 0.049, and <0.001, respectively). Transposon abundance was positively correlated with FOX and GEN resistance (*ρ* = 0.346 and 0.468; *p* = 0.031 and 0.003, respectively), whereas IS abundance was positively correlated with the overall phenotypic resistance burden (*ρ* = 0.319, *p* = 0.048). ICE/conjugation-associated feature abundance was negatively correlated with FFC resistance (*ρ* = −0.390, *p* = 0.014). Among ARGs, integron-associated features were positively correlated with *intI1* (*ρ* = 1.00, *p* < 0.001), ICE/conjugation-associated features were negatively correlated with *qnrA* (*ρ* = −0.54, *p* < 0.001), and transposon abundance was positively associated with *bla*_NDM-1_ positivity (ρ = 0.39, *p* < 0.05; Figure 7A,B). These unadjusted associations were interpreted as exploratory.

Correlations between MGE-associated features and VAGs are shown in Figure 7C. IS abundance was positively correlated with *A225_RS18975* and *PA3157* (*ρ* = 0.62 and 0.61, respectively; *p* < 0.001). Plasmid-associated feature abundance was positively correlated with multiple VAGs (*ρ* = 0.47, *p* < 0.01). ICE/conjugation-associated features were positively correlated with *aec14* and *EAMY_RS21105 (ρ =* 0.49 and 0.48, respectively; *p* < 0.01). Prophage-associated features were negatively correlated with *clbS* (*ρ* = −0.46, *p* < 0.01) and positively correlated with *fliC* (*ρ* = 0.47, *p* < 0.01), whereas transposon abundance was negatively correlated with *hpmB* (*ρ* = −0.39, *p* < 0.05). These exploratory findings suggest that the associations between MGE-associated features and VAGs varied according to the specific MGE category and gene examined.

### 3.8. Genomic Co-Occurrence of ARGs and VAGs with MGE-Associated Regions

**Figure 8:**
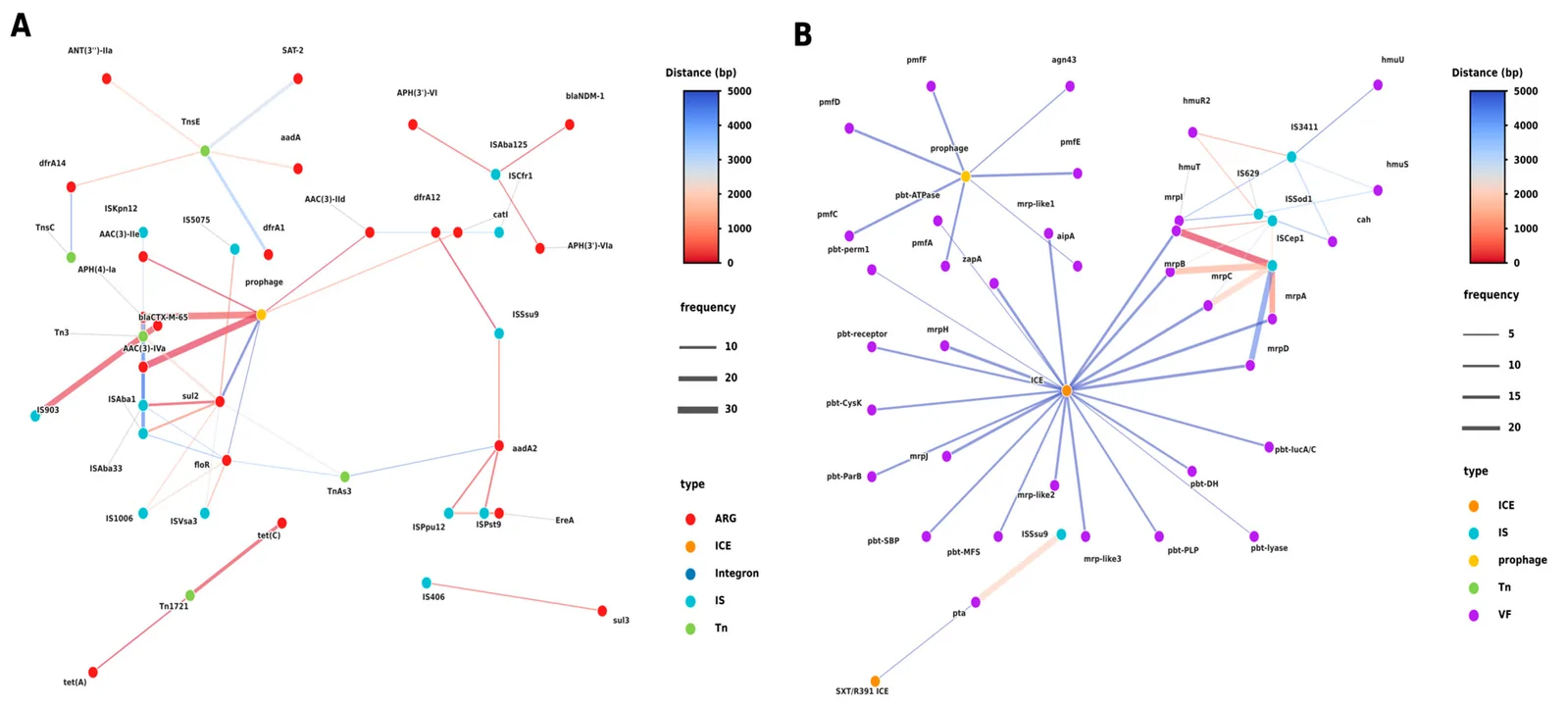
Annotation-based genomic co-occurrence networks between MGE-associated regions and resistance or virulence-associated genes. (**A**) Co-occurrence network of ARGs and MGE-associated regions. (**B**) Co-occurrence network of VAGs and MGE-associated regions. Each node represents an ARG, VAG, or putative MGE-associated region; node colors indicate genetic-element or gene categories. Edges indicate that the features met the defined same-contig co-occurrence criterion, namely, location within a putative MGE-associated region or within 5 kb of its boundary. Edge width is proportional to the co-occurrence frequency, and edge color represents the mean interfeature distance, ranging from red for shorter distances to blue for greater distances.

To investigate the genomic co-occurrence of MGE-associated regions with ARGs and VAGs, putative MGE-associated regions were delineated from genome annotations, and ARGs and VAGs located within or adjacent to these regions were examined. Across the 39 mink-derived *P. mirabilis* isolates, 739 ARG–MGE and 515 VAG–MGE co-occurrences were identified, indicating potential genetic associations between a subset of these genes and MGE-associated regions (Figure 8).

Within the ARG–MGE co-occurrence network, insertion sequence-, transposon-, and prophage-associated regions showed frequent links with multiple ARG classes. The most prominent association involved *IS903* and *bla*_CTX-M-65_, which met the defined co-occurrence criterion in 26 isolates. *ISAba125* and *bla*_NDM-1_ met the defined co-occurrence criterion in all three *bla*_NDM-1_-positive isolates. *Tn3*-associated regions co-occurred with the aminoglycoside-resistance genes *aac(3)-IVa* and *aph(4)-Ia* and the sulfonamide-resistance gene *sul2*, whereas *Tn1721* co-occurred with *tet(C)*. These patterns describe recurrent local genomic arrangements within the short-read assemblies. Prophage-associated regions also co-occurred with *aac(3)-IVa*, *aph(4)-Ia*, and *sul2* (Figure 8A).

Within the VAG–MGE co-occurrence network, putative ICE-, IS-, and prophage-associated regions co-occurred with multiple VAG categories. *ISEcp1* most frequently co-occurred with the MR/P fimbrial gene cluster, whereas *ISSsu9* co-occurred with the effector delivery-associated gene *pta*. Putative ICE-associated regions co-occurred with MR/P fimbrial genes, *zapA*, and several genes of the *pbt* iron-acquisition system. Prophage-associated regions most frequently co-occurred with the PMF fimbrial gene cluster (Figure 8B). Overall, these annotation-based co-occurrences represent same-contig proximity within draft assemblies rather than confirmed physical linkage within complete MGEs.

### 3.9. Population Structure of Mink-Derived Proteus mirabilis Isolates

**Figure 9:**
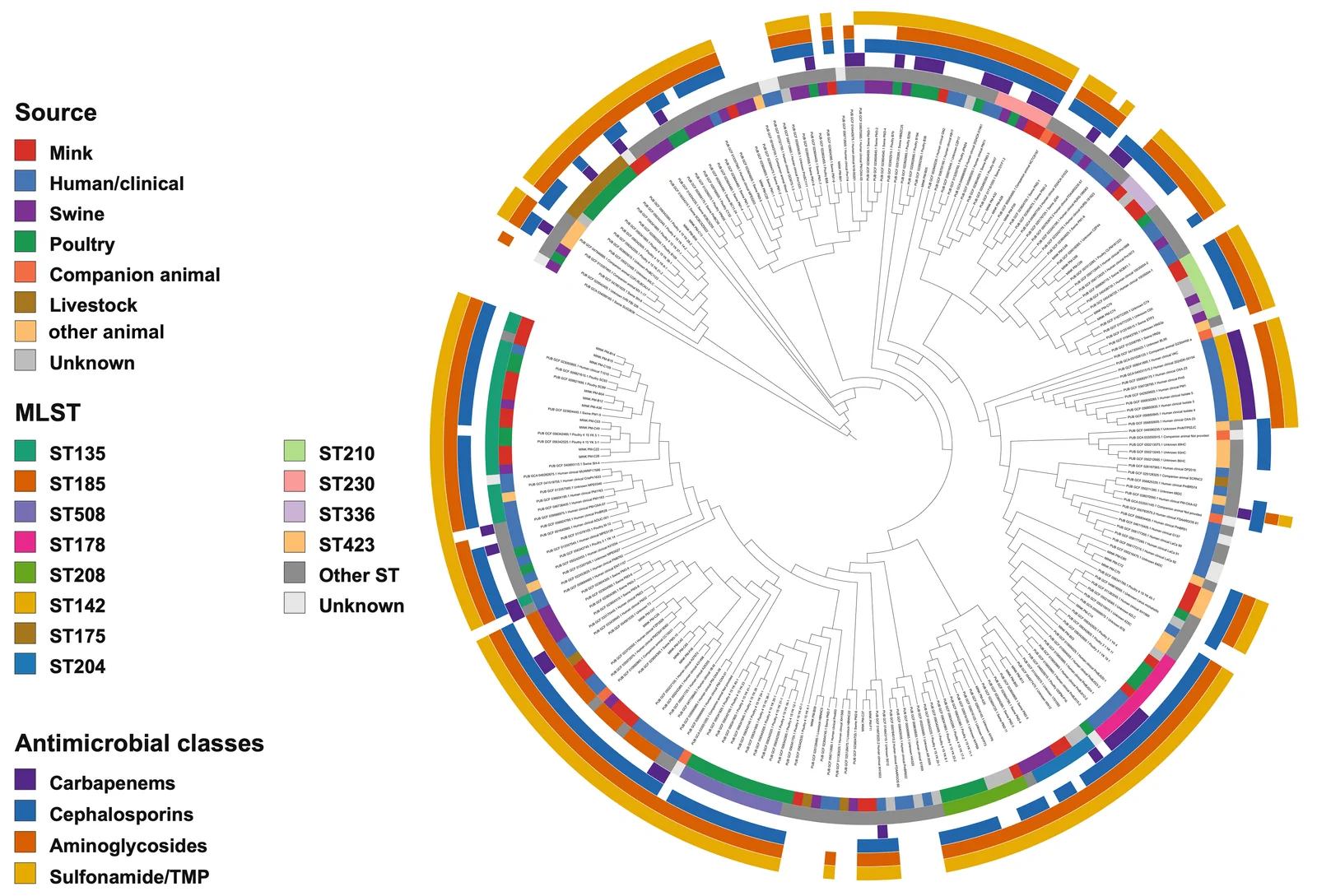
Population structure and distribution of major antimicrobial resistance gene classes among mink-derived *Proteus mirabilis* isolates and publicly available genomes. A Mash-based genomic distance clustering tree was constructed using the 39 mink-derived *P. mirabilis* isolates and 198 publicly available *P. mirabilis* genomes. From the innermost to the outermost ring, the annotation tracks represent isolate source, major sequence types (STs), and the presence of resistance genes associated with carbapenems, cephalosporins, aminoglycosides, and sulfonamides/trimethoprim, respectively. In the antimicrobial-class tracks, colored segments indicate the detection of at least one resistance gene assigned to the corresponding antimicrobial class, whereas white segments indicate that no such gene was detected. The publicly available genomes were included to provide a broader population genomic context for the mink-derived isolates.

To place the mink-derived *P. mirabilis* isolates within a broader genomic context, the 39 sequenced isolates were compared with 198 publicly available *P. mirabilis* genomes. The public genomes originated primarily from human/clinical, poultry, swine, companion animal, livestock, other animal, and unknown sources. Mash-based genomic distance clustering showed that the mink-derived isolates did not form a single exclusive cluster but were distributed across multiple genomic clusters. Their nearest neighboring public genomes in the distance-based tree were predominantly of human/clinical, swine, or poultry origin, indicating genomic similarity between the mink-derived isolates and *P. mirabilis* genomes from diverse host sources (Figure 9).

MLST analysis revealed considerable sequence-type diversity among the 39 mink-derived isolates. ST135 was the most common sequence type and comprised nine isolates, followed by ST185 with four isolates and ST336 and ST423 with three isolates each. Other detected sequence types included ST230, ST204, ST178, and ST210 (Supplementary Table S3). Several STs were shared by mink-derived isolates and publicly available genomes, demonstrating that the mink-derived isolates were not restricted to a single mink-associated lineage but occurred within multiple previously reported *P. mirabilis* genomic backgrounds.

The *bla*_CTX-M-65_-positive mink-derived isolates were distributed across multiple sequence types, including ST135, ST185, ST230, and ST423, as well as several less common STs. All mink-derived isolates within the ST135 cluster carried *bla*_CTX-M-65_. The three *bla*_NDM-1_-positive isolates comprised two ST230 isolates (PM-A28 and PM-A32), whereas no ST could be assigned to PM-B06 because its six-locus MLST profile was incomplete (Supplementary Table S3). Their nearest neighboring public genomes in the Mash-based tree originated from swine and poultry. Collectively, these findings indicate that the distribution of clinically important resistance genes among mink-derived *P. mirabilis* cannot be explained solely by the expansion of a single clone; rather, these genes occur across multiple genomic backgrounds.

## 4. Discussion

This study provides an integrated characterization of antimicrobial resistance, virulence-associated traits, and genomic diversity in *Proteus mirabilis* recovered from intensive mink farms in Shandong Province, China. Of the 68 isolates, 63 (92.6%) were multidrug resistant, and 39 (57.4%) were phenotypically confirmed as ESBL producers. Whole-genome analysis of the ESBL-positive subset revealed clinically important β-lactamase genes, substantial accessory-genome diversity, and abundant MGE-associated features. Both *bla*_CTX-M-65_- and *bla*_NDM-1_-positive isolates were distributed across multiple genomic lineages, with *bla*_CTX-M-65_ additionally detected in multiple sequence types. This distribution indicates that these genes were not restricted to a single clone but does not demonstrate horizontal gene transfer. Annotation-based analysis further showed same-contig co-occurrence between MGE-associated features and several resistance genes, notably *IS903*–*bla*_CTX-M-65_ and *ISAba125*–*bla*_NDM-1_. Lv et al. previously analyzed 53 *P. mirabilis* isolates collectively obtained from fox, raccoon dog, and mink farms in China and reported that 73.6% were resistant to at least three antimicrobial classes, with clinically important resistance genes, including *bla*_NDM_, also being detected [12]. However, the available study of fur-bearing animals combined isolates from different host species, leaving mink-derived ESBL-positive populations insufficiently characterized. The present study extends these observations by specifically examining mink-derived isolates and integrating resistance and virulence phenotyping with pangenome analysis, ARG and VAG profiling, characterization of MGE-associated features, and comparative population analysis of the ESBL-positive subset. These findings provide additional genomic evidence for evaluating antimicrobial-resistant *P. mirabilis* in fur-animal production systems.

Broad resistance across antimicrobial classes was a prominent feature of the mink-derived isolates. After doxycycline was excluded from the MDR calculation, 63 of the 68 isolates (92.6%) met the predefined MDR criterion. Resistance rates to trimethoprim–sulfamethoxazole, doxycycline, ampicillin, amikacin, and gentamicin each exceeded 79%. A previous study of *P. mirabilis* from broiler farms in Shandong Province similarly reported a 100% MDR rate, high resistance to trimethoprim–sulfamethoxazole, no resistance to aztreonam, and the occurrence of *bla*_NDM-1_ [37]. These similarities suggest that multidrug-resistant *P. mirabilis* may be maintained in different intensive animal-production settings, although they do not provide evidence of transmission between production systems. ESBL production was phenotypically confirmed in 39 isolates. Among the sequenced isolates, *bla*_CTX-M_ family genes were detected in 32 of 39 isolates, with *bla*_CTX-M-65_ being widely distributed. Nevertheless, seven phenotypically ESBL-positive isolates lacked detectable *bla*_CTX-M_ family genes, indicating that CTX-M-type enzymes did not fully account for the observed ESBL phenotypes. Other β-lactamase mechanisms, variation in gene expression, or limitations of sequence-based detection may have contributed to this genotype–phenotype discordance [3]. ESBL/AmpC-producing *P. mirabilis* carrying *bla*_CTX-M-65_ has also been reported in meat products and community-acquired urinary tract infections, demonstrating that this resistance genotype occurs in both food and human clinical sources [38]. Three isolates, PM-B06, PM-A28, and PM-A32, carried *bla*_NDM-1_. Similar *bla*_NDM-1_-positive *P. mirabilis* isolates have been reported in broilers and companion dogs [39,40], supporting the potential role of animal-derived *P. mirabilis* as a reservoir of this clinically important resistance determinant. However, the present study demonstrated only the genomic presence of *bla*_NDM-1_ and did not establish carbapenemase expression or horizontal transferability. In addition, because *P. mirabilis* has an intrinsic background of reduced susceptibility to some tetracyclines [19], the high doxycycline resistance rate observed here should not be attributed solely to acquired resistance genes. A limitation of this study is that farm-specific carriage data and detailed antimicrobial-use histories were unavailable; therefore, differences among individual farms could not be evaluated, and the observed AMR patterns cannot be directly attributed to specific antimicrobial exposures.

Biofilm formation, swarming motility, and urease activity are important phenotypes associated with adherence, surface migration, and persistent colonization by *P. mirabilis* [41]. Under the assay conditions used in this study, all 68 isolates were strong biofilm producers and urease positive, while 57 (83.8%) exhibited strong swarming motility. No significant differences in biofilm formation or swarming motility were observed between ESBL-positive and ESBL-negative isolates, indicating that these virulence-associated phenotypes were widely distributed but were not clearly associated with ESBL status. Similar coexistence of antimicrobial resistance, biofilm formation, and virulence-associated characteristics has been described in canine-derived *P. mirabilis* [42]. Genomic analysis of the 39 ESBL-positive isolates further revealed widespread genes associated with MR/P and PMF fimbriae, flagellar motility, and the proteobactin and HmuR2 iron-acquisition systems. MR/P and PMF fimbriae contribute to adherence, colonization, and biofilm architecture [43], whereas proteobactin- and heme-associated systems facilitate iron acquisition under nutrient-limited conditions [44,45]. Notably, although most sequenced isolates carried hemolysin-associated genes such as *hpmA* and *hpmB*, none of the 68 isolates exhibited detectable β-hemolytic activity under the conditions tested. This genotype–phenotype discordance highlights that the presence of virulence-associated genes does not necessarily result in expression of the corresponding phenotype and may depend on gene integrity, regulation, and environmental conditions. Therefore, the phenotypic and genomic characteristics identified here indicate potential traits relevant to colonization and virulence but should not be interpreted as direct evidence of pathogenicity in vivo.

MGE-associated features were widely distributed among the ESBL-positive isolates and showed category-specific relationships with antimicrobial resistance. In the exploratory analysis, prophage-associated feature abundance showed the strongest positive correlation with the overall phenotypic resistance burden (*ρ* = 0.615, unadjusted *p* < 0.001). Additional relationships reaching the unadjusted exploratory threshold included positive relationships of IS abundance with the overall phenotypic resistance burden and transposon abundance with FOX and GEN resistance, as well as a negative relationship between ICE/conjugation-associated feature abundance and FFC resistance. Associations between individual MGE categories and ARGs remained gene specific. Because no correction for multiple comparisons was applied and only 39 isolates were analyzed, these findings should be regarded as hypothesis-generating rather than firm evidence of association. At the genomic level, *IS903* and *bla*_CTX-M-65_ met the defined same-contig co-occurrence criterion in 26 isolates, whereas *ISAba125* and *bla*_NDM-1_ met this criterion in all three *bla*_NDM-1_-positive isolates. Previous studies have shown that *bla*_CTX-M_ and *bla*_NDM_ in *P. mirabilis* can occur within diverse genetic contexts, including transposon-associated structures, genomic islands, and SXT/R391-family ICEs [46]. *ISAba125*/*Tn125*-associated structures are also recognized as important genetic contexts for *bla*_NDM_ genes [47], providing biological plausibility for the genomic co-occurrence observed here. Selected VAGs were additionally located within or adjacent to putative ICE- and prophage-associated regions, consistent with previous reports of genomic associations between ARGs/VAGs and MGEs in animal-derived *P. mirabilis* [48]. ICEs retain the capacity for chromosomal integration, excision, and conjugative transfer [49], whereas prophages can influence bacterial genome evolution and virulence-related diversification [50]. Nevertheless, because the present analysis was based on short-read assemblies, the complete genetic structures, physical carriers, and transferability of these regions could not be established and would require validation by long-read sequencing. Collectively, these findings describe recurrent annotation-based genomic co-occurrence between MGE-associated regions and selected ARGs or VAGs in mink-derived *P. mirabilis*.

The substantial accessory-genome diversity observed among the ESBL-positive isolates provides an important genomic context for the heterogeneous distribution of ARGs and MGE-associated features. Pangenome analysis identified 7148 non-core gene families, and the continued increase in pangenome size indicated a trend toward an open pangenome. Large-scale comparative genomic studies have similarly revealed extensive accessory-genome diversity and lineage- and MGE-associated variation in resistance gene content in *P. mirabilis* [51,52], supporting accessory gene gain and loss as plausible contributors to the genomic heterogeneity observed among the mink-derived isolates. Population structure analysis further showed that the 39 ESBL-positive isolates were distributed across multiple STs and genomic clusters. Although ST135 was the most common sequence type, the population was not dominated by a single lineage. The *bla*_CTX-M-65_-positive isolates occurred in multiple genetic backgrounds, including ST135, ST185, ST230, and ST423, whereas the three *bla*_NDM-1_-positive isolates comprised two ST230 isolates and one isolate that remained untyped by MLST. Thus, these clinically important β-lactamase genes occurred in multiple genomic backgrounds rather than being restricted to a single clone. However, the mechanism underlying this distribution, including whether horizontal transfer occurred, could not be determined from the present short-read data. In the public-genome comparison, some mink-derived isolates clustered near genomes of human/clinical, swine, or poultry origin, indicating that isolates from different host sources may share similar genomic backgrounds. However, because the comparison was based on Mash distances and heterogeneous public metadata, these clustering patterns are insufficient to establish direct transmission or infer transmission direction [53].

From a One Health perspective, intensive, cage-based mink production may represent a relevant interface for AMR circulation. Animal feces and manure can serve as reservoirs of antimicrobial-resistant bacteria and ARGs in intensive production systems [9], and ESBL-producing bacteria have also been detected in mink feces and feed [11]. Moreover, a genomic study of LA-MRSA CC398 in Danish mink production identified epidemiological links among mink, mink feed, pigs, and occupationally exposed farm workers [54]. Although these findings concern bacterial species other than *P. mirabilis*, they illustrate potential connections among animals, farm-associated materials, the environment, and humans within mink production systems. However, detailed antimicrobial-use histories and samples of feed, manure, wastewater, the farm environment, farm workers, and other animal populations were not collected in the present study. These possible connections should therefore be regarded as One Health hypotheses requiring targeted investigation rather than demonstrated transmission pathways.

Several limitations of this study should be acknowledged. The six farms included in this study were large-scale commercial mink farms in Shandong Province. Nevertheless, the sampling was restricted to these farms, and the findings should be extrapolated cautiously to smaller farms, other production systems, or other geographic regions. In addition, WGS was performed only on the 39 ESBL-positive isolates; therefore, the genomic findings characterize this selected subset and should not be generalized to all 68 *P. mirabilis* isolates recovered in this study.

## 5. Conclusions

This study characterized the antimicrobial resistance, virulence-associated traits, and genomic features of *Proteus mirabilis* isolates recovered from intensive mink farms in Shandong Province, China. Of the 68 isolates, 63 (92.6%) were multidrug resistant, and 39 (57.4%) were phenotypically ESBL positive. Whole-genome analysis of the ESBL-positive subset revealed the widespread occurrence of clinically important β-lactamase genes, particularly *bla*_CTX-M-65_, together with the detection of *bla*_NDM-1_ in three isolates. The *bla*_CTX-M-65_-positive isolates occurred across multiple sequence types, whereas the *bla*_NDM-1_-positive isolates occurred in more than one genomic background, suggesting that their distribution was not attributable solely to expansion of a single clone. Recurrent same-contig co-occurrence was observed between *IS903* and *bla*_CTX-M-65_ and between *ISAba125* and *bla*_NDM-1_; however, complete genetic structures, physical carriers, and transferability could not be established from the short-read assemblies. Biofilm formation, urease activity, and swarming motility were common among the isolates, while the sequenced subset exhibited substantial accessory-genome diversity and widely distributed virulence-associated genes. Collectively, these findings identify intensive mink production as an understudied setting harboring multidrug-resistant, ESBL-producing *P. mirabilis* and support the inclusion of fur-animal production systems in One Health antimicrobial resistance surveillance.

## Figures and Tables

**Table 1:** Minimum inhibitory concentration ranges, MIC_50_ and MIC_90_ values, and resistance rates of 68 mink-derived *Proteus mirabilis* isolates against 11 antimicrobial agents.

Antimicrobial Agent	Resistance, *n* (%)	MIC_50_ (μg/mL)	MIC_90_ (μg/mL)	MIC Range
Doxycycline (DO)	65 (95.6)	>32	>32	2–>32
Imipenem (IPM)	11 (16.2)	2	16	0.5–32
Cefepime (FEP)	13 (19.1)	2	16	0.25–32
Gentamicin (GEN)	54 (79.4)	32	>32	1–>32
Amikacin (AMK)	61 (89.7)	32	>32	8–>32
Enrofloxacin (ENR)	35 (51.5)	4	>8	0.125–>8
Cefoxitin (FOX)	6 (8.8)	4	16	1–32
Ampicillin (AM)	63 (92.6)	32	>32	1–>32
Florfenicol (FFC)	37 (54.4)	32	>32	1–>32
Trimethoprim-sulfamethoxazole (SXT)	66 (97.1)	≥4/76	≥4/76	≤2/38–≥4/76
Aztreonam (ATM)	0 (0.0)	2	4	1–4

## Data Availability

The draft genome assemblies generated in this study are available in NCBI GenBank under BioProject accession number PRJNA1494192. Isolate-specific GenBank WGS accession numbers are listed in Supplementary Table S3.

## Supplementary Materials

The following supporting information can be downloaded at: https://www.mdpi.com/article/10.3390/antibiotics15090933/s1, Table S1: Phenotypic resistance patterns of 68 mink-derived *Proteus mirabilis* isolates; Table S2: GenBank accession numbers, host sources, assembly levels, and sequence types of the 198 publicly available *Proteus mirabilis* genomes included in the comparative analysis; Table S3: Sampling metadata and sequence types of the 39 ESBL-producing mink-derived *P. mirabilis* isolates subjected to whole-genome sequencing; Table S4: Minimum inhibitory concentration (MIC) interpretive criteria and antimicrobial-category assignments used for antimicrobial susceptibility testing and MDR classification of mink-derived *Proteus mirabilis* isolates; Table S5: Average nucleotide identity (ANI) analysis of the 39 sequenced mink-derived *P. mirabilis* isolates.
