# Supplementary material for: Antimicrobial Resistance and Genomic Characteristics of ESBL-Producing Proteus mirabilis from Farmed Mink in Shandong Province, China
Source: Antibiotics (Basel). 2026 Sep 20;15(9):933. doi: 10.3390/antibiotics15090933 (PMC13603337; doi:10.3390/antibiotics15090933)
Supplement: Supplementary file 1 [file antibiotics-15-00933-s001.zip › antibiotics-4528661-supplementary.pdf]

Supplementary Table S1. Phenotypic resistance patterns of 68 mink-derived *Proteus mirabilis* isolates.

Isolate ID	ESBL phenotype	Source	Region	Phenotypic resistance pattern	No. of resistant agents
PM-A28	Positive	Feces	Weihai	DO–FEP–GEN–AMK–ENR–IPM–FOX–AM–FFC–SXT	10
PM-A29	Negative	Feces	Weihai	DO–FEP–GEN–AMK–ENR–IPM–FOX–AM–FFC–SXT	10
PM-A31	Negative	Feces	Weihai	DO–GEN–AMK–ENR–IPM–AM–FFC–SXT	8
PM-A32	Positive	Feces	Weihai	DO–FEP–GEN–AMK–ENR–IPM–FOX–AM–FFC–SXT	10
PM-A35	Positive	Feces	Weihai	GEN–AMK–AM–FFC–SXT	5
PM-A36	Positive	Feces	Weihai	DO–GEN–AMK–ENR–AM–SXT	6
PM-A37	Negative	Feces	Weihai	DO–GEN–AMK–ENR–IPM–FFC–SXT	7
PM-B01	Positive	Feces	Weihai	DO–GEN–AMK–ENR–AM–FFC–SXT	7
PM-B02	Positive	Feces	Weihai	DO–FEP–GEN–AMK–ENR–AM–SXT	7
PM-B03	Positive	Feces	Weihai	DO–AMK–ENR–FOX–AM–FFC–SXT	7
PM-B04	Positive	Feces	Weihai	DO–FEP–GEN–AMK–ENR–AM–FFC–SXT	8
PM-B05	Positive	Feces	Weihai	DO–GEN–AMK–IPM–AM–FFC–SXT	7
PM-B06	Positive	Feces	Weihai	DO–GEN–AMK–ENR–IPM–AM–SXT	7
PM-B09	Positive	Feces	Weihai	DO–AMK–ENR–IPM–AM–SXT	6
PM-B10	Negative	Feces	Weihai	DO–GEN–AMK–ENR–IPM–AM–FFC–SXT	8
PM-B12	Positive	Feces	Weihai	DO–GEN–AMK–ENR–IPM–AM–FFC–SXT	8
PM-B13	Positive	Feces	Weihai	DO–FEP–GEN–AMK–ENR–AM–FFC–SXT	8
PM-B14	Positive	Feces	Weihai	DO–FEP–GEN–AMK–ENR–AM–FFC–SXT	8
PM-B15	Positive	Feces	Weihai	DO–FEP–GEN–AMK–ENR–AM–FFC–SXT	8
PM-C03	Positive	Intestinal contents	Weihai	DO–GEN–ENR–AM–SXT	5
PM-C06	Positive	Intestinal contents	Weihai	DO–GEN–AMK–AM–FFC–SXT	6
PM-C07	Positive	Intestinal contents	Weihai	DO–GEN–AMK–AM–FFC–SXT	6
PM-C08	Negative	Intestinal contents	Weihai	DO–GEN–AMK–AM–FFC–SXT	6
PM-C09	Negative	Intestinal contents	Weihai	DO–GEN–AMK–AM	4
PM-C12	Negative	Intestinal contents	Weihai	DO–FEP–GEN–AMK–ENR–AM–FFC–SXT	8
PM-C13	Positive	Intestinal contents	Weifang	DO–GEN–AMK–AM–SXT	5

Isolate ID	ESBL phenotype	Source	Region	Phenotypic resistance pattern	No. of resistant agents
PM-C14	Positive	Intestinal contents	Weifang	DO–ENR–FOX–AM–FFC–SXT	6
PM-C17	Negative	Intestinal contents	Weifang	DO–AMK–AM–SXT	4
PM-C20	Positive	Intestinal contents	Weifang	DO–GEN–AM–SXT	4
PM-C22	Positive	Intestinal contents	Weifang	DO–GEN–ENR–AM–SXT	5
PM-C24	Negative	Intestinal contents	Weifang	DO–AMK–AM	3
PM-C25	Negative	Intestinal contents	Weifang	DO–GEN–AMK–ENR–AM–FFC–SXT	7
PM-C26	Positive	Intestinal contents	Weifang	DO–FEP–GEN–AMK–ENR–AM–FFC–SXT	8
PM-C27	Negative	Intestinal contents	Weifang	DO–GEN–AMK–AM–FFC–SXT	6
PM-C28	Positive	Intestinal contents	Weifang	DO–FEP–GEN–AMK–ENR–FOX–AM–FFC–SXT	9
PM-C32	Negative	Intestinal contents	Weifang	DO–GEN–AMK–AM–SXT	5
PM-C36	Negative	Intestinal contents	Weifang	DO–GEN–AMK–SXT	4
PM-C37	Positive	Intestinal contents	Weifang	DO–GEN–AMK–AM–FFC–SXT	6
PM-C38	Negative	Intestinal contents	Weifang	DO–GEN–AMK–AM–SXT	5
PM-C42	Positive	Intestinal contents	Weifang	DO–GEN–AMK–AM–SXT	5
PM-C44	Negative	Intestinal contents	Weifang	DO–GEN–AMK–AM–FFC–SXT	6
PM-C45	Negative	Intestinal contents	Weifang	DO–GEN–AMK–AM–SXT	5
PM-C48	Positive	Intestinal contents	Weifang	DO–GEN–AMK–AM–SXT	5
PM-C49	Positive	Intestinal contents	Weifang	DO–GEN–AMK–ENR–AM–SXT	6
PM-C50	Negative	Intestinal contents	Weifang	DO–AM–SXT	3
PM-C51	Negative	Intestinal contents	Weifang	DO–GEN–FFC–SXT	4
PM-C56	Negative	Intestinal contents	Weifang	DO–AMK–SXT	3
PM-C58	Negative	Intestinal contents	Weifang	DO–GEN–ENR–AM–FFC–SXT	6
PM-C62	Negative	Intestinal contents	Weifang	DO–GEN–AMK–ENR–AM–FFC–SXT	7
PM-C63	Negative	Intestinal contents	Weifang	DO–AMK–AM–FFC–SXT	5
PM-C65	Positive	Intestinal contents	Weifang	DO–GEN–AMK–AM–SXT	5
PM-C68	Positive	Intestinal contents	Weifang	DO–GEN–AMK–AM–FFC–SXT	6
PM-C70	Positive	Intestinal contents	Weifang	DO–AMK–ENR–AM–SXT	5

Isolate ID	ESBL phenotype	Source	Region	Phenotypic resistance pattern	No. of resistant agents
PM-C71	Negative	Intestinal contents	Weifang	DO–AMK–AM–SXT	4
PM-C72	Positive	Intestinal contents	Weifang	DO–AMK–ENR–AM–SXT	5
PM-C74	Positive	Intestinal contents	Weifang	DO–GEN–AMK–ENR–AM–FFC–SXT	7
PM-C76	Negative	Intestinal contents	Weifang	GEN–AMK–AM–SXT	4
PM-C79	Positive	Intestinal contents	Weifang	DO–GEN–AMK–ENR–AM–FFC–SXT	7
PM-C80	Positive	Intestinal contents	Linyi	DO–AMK–ENR–AM–SXT	5
PM-C81	Negative	Intestinal contents	Linyi	DO–AMK–AM–FFC–SXT	5
PM-C88	Negative	Intestinal contents	Linyi	DO–AMK–AM–SXT	4
PM-C94	Negative	Intestinal contents	Linyi	DO–GEN–AMK–ENR–SXT	5
PM-C106	Negative	Intestinal contents	Linyi	GEN–AMK–AM–SXT	4
PM-C109	Positive	Intestinal contents	Linyi	DO–FEP–GEN–AMK–ENR–AM–SXT	7
PM-F06	Positive	Feces	Yantai	DO–GEN–AMK–AM–SXT	5
PM-F09	Positive	Feces	Yantai	DO–GEN–AMK–AM–FFC–SXT	6
PM-F11	Positive	Feces	Yantai	DO–FEP–GEN–AMK–ENR–AM–FFC–SXT	8
PM-F12	Negative	Feces	Yantai	DO–GEN–AMK–IPM–AM–FFC–SXT	7

Note: Abbreviations: DO, doxycycline; FEP, cefepime; GEN, gentamicin; AMK, amikacin; ENR, enrofloxacin; IPM, imipenem; FOX, cefoxitin; AM, ampicillin; FFC, florfenicol; SXT, trimethoprim-sulfamethoxazole. Doxycycline was retained in the phenotypic resistance profiles and resistant-agent counts but was excluded from MDR classification.

Supplementary Table S2. GenBank accession numbers, host sources, assembly levels, and sequence types of the 198 publicly available *Proteus mirabilis* genomes included in the comparative analysis.

GenBank accession	Strain	Host/source	Assembly level	ST
GCA_034099165.1	SUG3636	Swine	Contig	Unknown
GCF_029542405.1	IVRI/FBI/326	Gallus gallus (Poultry)	Complete Genome	ST445
GCF_047801625.1	SH-8	Swine	Complete Genome	ST132
GCF_013357465.1	M3-1-17	Cacatua galerita (Other animal)	Complete Genome	ST132
GCF_047946885.1	COFI-RLBCAU-II	Labeo catla (Other animal)	Complete Genome	ST132
GCF_050212495.1	84LC	Larus michahellis (Other animal)	Complete Genome	ST162
GCF_022802535.1	B109	chicken (Poultry)	Scaffold	ST175
GCF_056341885.1	4_15_YK_37-1	chicken (Poultry)	Scaffold	ST175
GCF_056342085.1	4_15_YK_28-2	chicken (Poultry)	Scaffold	ST175
GCF_056342345.1	4_15_YK_14-2	chicken (Poultry)	Scaffold	ST175
GCF_003855615.1	PmBC1123	Unknown	Complete Genome	ST175
GCF_056342205.1	4_15_YK_21-2	chicken (Poultry)	Scaffold	ST175
GCF_056341865.1	4_15_YK_38-1	chicken (Poultry)	Scaffold	ST175
GCF_056342505.1	4_15_YK_6-1	chicken (Poultry)	Scaffold	ST175
GCF_018334315.1	SCBCSZ5522	Swine	Contig	ST177
GCF_003856175.1	PmBC55	Swine	Contig	ST177
GCF_018334235.1	SCBCSZ55	Swine	Contig	ST177
GCF_022802455.1	B58	chicken (Poultry)	Scaffold	ST187
GCF_023604395.1	PM1-6	Swine	Scaffold	ST300
GCF_038070585.1	PM-OXA-05	Homo sapiens (Human/clinical)	Complete Genome	ST343
GCF_023604655.1	PM3-1	Swine	Scaffold	ST302
GCF_023604545.1	PM3-4	Swine	Scaffold	ST302
GCF_023604645.1	PM3-2	Swine	Scaffold	ST302
GCF_010442675.1	N18-00201	Homo sapiens (Human/clinical)	Complete Genome	ST195
GCF_056712605.1	Pm114	Homo sapiens (Human/clinical)	Complete Genome	Unknown
GCF_003855635.1	PmSC1111	Unknown	Complete Genome	ST176

GenBank accession	Strain	Host/source	Assembly level	ST
GCF_023604425.1	PM1-7	Swine	Scaffold	ST136
GCF_023604555.1	PM1-9	Swine	Scaffold	ST136
GCF_001643755.1	Wood	Lucilia sericata (Other animal)	Contig	ST499
GCF_056712665.1	Pm1325	Homo sapiens (Human/clinical)	Complete Genome	Unknown
GCF_057221795.1	GCM75-1-3	Homo sapiens (Human/clinical)	Complete Genome	Unknown
GCF_013256075.1	YPM35	duck (Poultry)	Complete Genome	ST154
GCF_002944495.1	BC11-24	pig (Swine)	Complete Genome	ST154
GCF_028768505.1	CY32	chicken (Poultry)	Complete Genome	ST154
GCF_023604465.1	PM1-3	Swine	Scaffold	ST301
GCF_023604485.1	PM1-4	Swine	Scaffold	ST301
GCF_013357565.1	MPE5203	Homo sapiens (Human/clinical)	Complete Genome	ST215
GCF_023604605.1	PM3-5	Swine	Scaffold	ST271
GCF_023604475.1	PM1-2	Swine	Scaffold	ST271
GCF_023604855.1	PM1-1	Swine	Scaffold	ST271
GCF_022802515.1	B79	chicken (Poultry)	Scaffold	ST291
GCF_054977475.1	WN12	Unknown	Complete Genome	ST204
GCF_023604805.1	PM2-5	Swine	Scaffold	ST204
GCF_023604285.1	PM2-3	Swine	Scaffold	ST204
GCF_023604785.1	PM2-4	Swine	Scaffold	ST204
GCF_011045575.1	1701092	Unknown	Complete Genome	ST204
GCF_046529215.1	GDPM-P16	chicken (Poultry)	Complete Genome	Unknown
GCF_023604725.1	PM2-11	Swine	Scaffold	ST305
GCF_056342385.1	4_15_YK_11-1	chicken (Poultry)	Scaffold	ST208
GCF_056342285.1	4_15_YK_17-2	chicken (Poultry)	Scaffold	ST208
GCF_056341965.1	4_15_YK_33-2	chicken (Poultry)	Scaffold	ST208
GCF_056342225.1	4_15_YK_20-1	chicken (Poultry)	Scaffold	ST208
GCF_056342405.1	4_15_YK_8-1	chicken (Poultry)	Scaffold	ST208
GCF_026016145.1	NYP6	Unknown	Complete Genome	ST208

GenBank accession	Strain	Host/source	Assembly level	ST
GCF_026016105.1	NYP69	Unknown	Complete Genome	ST208
GCF_026016125.1	NYP73	Unknown	Complete Genome	ST208
GCF_002055685.1	AR_0059	missing (Unknown)	Complete Genome	ST114
GCF_000784015.2	FDAARGOS_60	Homo sapiens (Human/clinical)	Complete Genome	ST96
GCF_056825035.1	PmBR622	Homo sapiens (Human/clinical)	Complete Genome	ST96
GCF_000069965.1	HI4320	Unknown	Complete Genome	ST90
GCF_008630655.1	S1959	Homo sapiens (Human/clinical)	Complete Genome	ST1
GCF_014843115.1	S012	Unknown	Complete Genome	ST188
GCF_018972025.2	XH1653	Homo sapiens (Human/clinical)	Complete Genome	ST188
GCF_023604745.1	PM2-7	Swine	Scaffold	ST519
GCF_056712685.1	Pm493	Homo sapiens (Human/clinical)	Complete Genome	ST235
GCF_056342535.1	4_15_YK_4-1	chicken (Poultry)	Scaffold	ST818
GCF_056341705.1	4_15_YK_47-1	chicken (Poultry)	Scaffold	ST508
GCF_056342005.1	4_15_YK_30-1	chicken (Poultry)	Scaffold	ST508
GCF_056342025.1	4_15_YK_31-1	chicken (Poultry)	Scaffold	ST508
GCF_056342305.1	4_15_YK_16-1	chicken (Poultry)	Scaffold	ST508
GCF_056342365.1	4_15_YK_12-1	chicken (Poultry)	Scaffold	ST508
GCF_056341905.1	4_15_YK_36-1	chicken (Poultry)	Scaffold	ST508
GCF_056341845.1	4_15_YK_39	chicken (Poultry)	Scaffold	ST508
GCF_056342145.1	4_15_YK_24-1	chicken (Poultry)	Scaffold	ST508
GCF_056341805.1	4_15_YK_41-1	chicken (Poultry)	Scaffold	ST508
GCF_056341825.1	4_15_YK_40-1	chicken (Poultry)	Scaffold	ST508
GCF_056342165.1	4_15_YK_23	chicken (Poultry)	Scaffold	ST508
GCF_025128485.1	HBNNC5	dairy cow (Livestock)	Contig	ST275
GCF_009684665.1	ENT1157	Homo sapiens (Human/clinical)	Complete Genome	ST192
GCF_022453625.1	PM8762	Homo sapiens (Human/clinical)	Complete Genome	ST135
GCF_013357505.1	MPE0027	Panthera tigris Amoyensis facel (Other animal)	Complete Genome	ST140
GCF_050242455.1	A31034	Homo sapiens (Human/clinical)	Complete Genome	ST207

GenBank accession	Strain	Host/source	Assembly level	ST
GCF_056342745.1	3_1_YK_14	chicken (Poultry)	Scaffold	ST207
GCF_011078105.1	M-12	Chicken (Poultry)	Scaffold	ST207
GCF_013357545.1	MPE5139	Homo sapiens (Human/clinical)	Complete Genome	ST207
GCF_040893115.1	SH-4	Swine	Complete Genome	ST135
GCF_009821615.1	SC93	chicken (Poultry)	Scaffold	ST135
GCF_009821695.1	SC89	chicken (Poultry)	Scaffold	ST135
GCF_023093855.1	T1010	Homo sapiens (Human/clinical)	Complete Genome	ST135
GCF_023604445.1	PM1-5	Swine	Scaffold	ST135
GCF_056342485.1	4_15_YK_5_1	chicken (Poultry)	Scaffold	ST135
GCF_056342525.1	4_15_YK_3-1	chicken (Poultry)	Scaffold	ST135
GCF_001640985.1	AOUC-001	Homo sapiens (Human/clinical)	Complete Genome	ST93
GCF_039566975.1	PM-OXA-07	Homo sapiens (Human/clinical)	Complete Genome	ST461
GCF_056824785.1	PmBR28	Homo sapiens (Human/clinical)	Complete Genome	ST93
GCF_036604195.1	PM1163	Homo sapiens (Human/clinical)	Complete Genome	ST135
GCF_046736405.1	PM1163	patient (Human/clinical)	Complete Genome	ST135
GCF_013357585.1	MPE0346	Malayan pangolin (Other animal)	Complete Genome	ST135
GCA_048282675.1	MUWRP17566	Homo sapiens (Human/clinical)	Complete Genome	Unknown
GCF_041519755.1	CriePir1633	Homo sapiens (Human/clinical)	Complete Genome	ST135
GCA_033551255.1	Not provided	Felis catus (Companion animal)	Contig	Unknown
GCF_039566985.1	PM-OXA-06	Homo sapiens (Human/clinical)	Complete Genome	ST479
GCF_039566995.1	PM-OXA-01	Homo sapiens (Human/clinical)	Complete Genome	ST479
GCF_047758645.1	1B-M	Homo sapiens (Human/clinical)	Complete Genome	ST185
GCF_050241725.1	A25355	Homo sapiens (Human/clinical)	Complete Genome	ST185
GCF_050237105.1	A27672	Homo sapiens (Human/clinical)	Complete Genome	ST185
GCF_050242385.1	A31498	Homo sapiens (Human/clinical)	Complete Genome	ST185
GCF_054901035.1	T3	Bos taurus (Livestock)	Complete Genome	ST185
GCF_023604315.1	PM3-8	Swine	Scaffold	ST185
GCF_023604385.1	PM3-7	Swine	Scaffold	ST185

GenBank accession	Strain	Host/source	Assembly level	ST
GCF_023604305.1	PM3-9	Swine	Scaffold	ST185
GCF_023604565.1	PM3-6	Swine	Scaffold	ST185
GCF_033170445.1	PM23	Homo sapiens (Human/clinical)	Complete Genome	ST185
GCF_033439945.1	PM32	Homo sapiens (Human/clinical)	Complete Genome	ST185
GCF_023702555.1	FZP2826	Homo sapiens (Human/clinical)	Complete Genome	ST185
GCF_035972075.1	PM230418060	patient (Human/clinical)	Complete Genome	ST185
GCF_010692865.1	CC15031	dog (Companion animal)	Complete Genome	ST203
GCF_023604365.1	PM3-10	Swine	Scaffold	ST185
GCF_023604755.1	PM2-6	Swine	Scaffold	ST306
GCF_011383025.1	XH1568	Homo sapiens (Human/clinical)	Complete Genome	ST209
GCF_025128475.1	HBNNC22	dairy cow (Livestock)	Contig	ST265
GCF_025128395.1	HBSZC25	Swine	Contig	ST321
GCF_022802665.1	B250	chicken (Poultry)	Scaffold	ST288
GCF_022802395.1	B38	chicken (Poultry)	Scaffold	ST288
GCF_022802695.1	B194	chicken (Poultry)	Scaffold	ST288
GCF_002945235.1	GN2	Homo sapiens (Human/clinical)	Complete Genome	ST155
GCF_008705195.1	K817	Homo sapiens (Human/clinical)	Complete Genome	ST190
GCF_013255765.1	JPM24	Chicken (Poultry)	Complete Genome	ST190
GCF_026016045.1	CZP17	Unknown	Complete Genome	ST190
GCA_046688905.2	2024CK-01961	Homo sapiens (Human/clinical)	Complete Genome	ST556
GCF_045689305.1	PM11	Homo sapiens (Human/clinical)	Complete Genome	ST230
GCF_023604625.1	PM3-3	Swine	Scaffold	ST230
GCF_017161055.1	DY.F1.2	Swine	Complete Genome	ST230
GCF_025490355.1	W47	chicken (Poultry)	Complete Genome	ST230
GCF_023604825.1	PM1-8	Swine	Scaffold	ST307
GCF_026016065.1	CZP44	Unknown	Complete Genome	ST336
GCF_016772335.1	C55	Unknown	Complete Genome	ST210
GCF_016772355.1	C74	Unknown	Complete Genome	ST210

GenBank accession	Strain	Host/source	Assembly level	ST
GCF_012516515.1	STP3	Swine	Complete Genome	ST210
GCF_013358795.1	HN2p	Swine	Complete Genome	ST210
GCF_019443785.1	HNS2p	Unknown	Complete Genome	ST210
GCF_009806715.1	SCBX1.1	Swine	Complete Genome	ST170
GCF_040436725.1	10035004-1	Homo sapiens (Human/clinical)	Complete Genome	ST170
GCF_040436735.1	10035004-2	Homo sapiens (Human/clinical)	Complete Genome	ST170
GCF_007012285.1	CLPM181223	Duck (Poultry)	Chromosome	ST186
GCF_056712625.1	Pm1673	Homo sapiens (Human/clinical)	Complete Genome	ST186
GCF_056712645.1	Pm1668	Homo sapiens (Human/clinical)	Complete Genome	ST186
GCA_040897705.2	2024CK-01032	Homo sapiens (Human/clinical)	Complete Genome	ST157
GCF_025176725.1	JZ49	Swine	Scaffold	ST228
GCF_000783875.2	FDAARGOS_67	Homo sapiens (Human/clinical)	Complete Genome	ST101
GCF_023242175.1	HURS-181823	Homo sapiens (Human/clinical)	Complete Genome	ST298
GCF_023242195.1	HURS-186083	Homo sapiens (Human/clinical)	Complete Genome	ST298
GCF_900454995.1	NCTC6197	Dog (Companion animal)	Contig	ST171
GCF_023604675.1	PM2-2	Swine	Scaffold	ST218
GCF_023604705.1	PM2-1	Swine	Scaffold	ST218
GCF_047305425.1	BL95	Mus musculus strain C57BL/6 (Other animal)	Complete Genome	ST146
GCA_031026125.1	G2304450_a	canine (Companion animal)	Contig	Unknown
GCF_008041895.1	VAC	Homo sapiens (Human/clinical)	Complete Genome	ST142
GCA_040031515.2	2024DK-00154	Homo sapiens (Human/clinical)	Complete Genome	ST142
GCF_056829175.1	OXA-23	Homo sapiens (Human/clinical)	Complete Genome	ST142
GCF_039728795.1	Pm5	Homo sapiens (Human/clinical)	Complete Genome	ST142
GCF_042929925.1	PM1	Homo sapiens (Human/clinical)	Complete Genome	ST142
GCF_056830285.1	Isolate 5	Homo sapiens (Human/clinical)	Complete Genome	ST142
GCF_056850835.1	Isolate 3	Homo sapiens (Human/clinical)	Complete Genome	ST142
GCF_056850605.1	OXA-23	Homo sapiens (Human/clinical)	Complete Genome	ST142
GCF_056850845.1	Isolate 4	Homo sapiens (Human/clinical)	Complete Genome	ST142

GenBank accession	Strain	Host/source	Assembly level	ST
GCF_049390235.1	PmMTP02JC	Larus michahellis (Other animal)	Complete Genome	ST163
GCA_033550915.1	Not provided	Felis catus (Companion animal)	Contig	Unknown
GCF_050213075.1	89HC	Larus michahellis (Other animal)	Complete Genome	ST130
GCF_050212685.1	86HC	Larus michahellis (Other animal)	Complete Genome	ST130
GCF_050213245.1	93HC	Larus michahellis (Other animal)	Complete Genome	ST130
GCF_056341765.1	4_15_YK_45-1	chicken (Poultry)	Scaffold	ST819
GCF_050215915.1	42SC	Larus michahellis (Other animal)	Complete Genome	ST115
GCF_050216065.1	62LC	Larus michahellis (Other animal)	Complete Genome	ST115
GCF_011383045.1	XH1569	Homo sapiens (Human/clinical)	Complete Genome	ST115
GCF_049818595.1	Larus michahellis	Unknown	Complete Genome	ST115
GCA_055699075.1	R76	Unknown	Complete Genome	ST178
GCF_056342925.1	3_1_YK_4	chicken (Poultry)	Scaffold	ST178
GCF_056342665.1	3_1_YK_19_1	chicken (Poultry)	Scaffold	ST178
GCF_056342805.1	3_1_YK_11	chicken (Poultry)	Scaffold	ST178
GCF_015694025.1	PmBJ020-1	Homo sapiens (Human/clinical)	Complete Genome	ST178
GCF_015694005.1	PmBJ023-2	Homo sapiens (Human/clinical)	Complete Genome	ST178
GCF_015693985.1	PmBJ024-1	Homo sapiens (Human/clinical)	Complete Genome	ST178
GCF_015693865.1	PmBJ015-2	Homo sapiens (Human/clinical)	Complete Genome	ST178
GCF_015693965.1	PmBJ012-2	Homo sapiens (Human/clinical)	Complete Genome	ST178
GCF_050216475.1	64DC	Larus michahellis (Other animal)	Complete Genome	ST160
GCF_025128325.1	SCRNC2	beef cattle (Livestock)	Contig	ST107
GCF_026167565.1	DP2019	Homo sapiens (Human/clinical)	Complete Genome	ST337
GCA_033551145.1	Not provided	Felis catus (Companion animal)	Contig	Unknown
GCF_038070595.1	PM-OXA-02	Homo sapiens (Human/clinical)	Complete Genome	ST484
GCF_050211395.1	68DC	Larus michahellis (Other animal)	Complete Genome	ST116
GCF_056825335.1	PmBR574	Homo sapiens (Human/clinical)	Complete Genome	ST717
GCF_000783575.2	FDAARGOS_81	Homo sapiens (Human/clinical)	Complete Genome	ST99
GCF_056834405.1	PmBR51	Homo sapiens (Human/clinical)	Complete Genome	Unknown

GenBank accession	Strain	Host/source	Assembly level	ST
GCF_046115405.1	G137	Homo sapiens (Human/clinical)	Complete Genome	ST565
GCF_058177355.1	LaCa_90	Homo sapiens (Human/clinical)	Complete Genome	ST627
GCF_058177215.1	LaCa_92	Homo sapiens (Human/clinical)	Complete Genome	Unknown
GCF_058177285.1	LaCa_91	Homo sapiens (Human/clinical)	Complete Genome	Unknown

Supplementary Table S3. Sampling metadata, sequence types, and GenBank WGS accession numbers of the 39 mink-derived *Proteus mirabilis* isolates subjected to whole-genome sequencing.

Isolate ID	Sampling location	Sample type	Sequence type (ST)	GenBank WGS accession
PM-A28	Weihai	Feces	ST230	JCBAFK000000000
PM-A32	Weihai	Feces	ST230	JCBAFJ000000000
PM-A35	Weihai	Feces	ST208	JCBAFI000000000
PM-A36	Weihai	Feces	ST135	JCBAFH000000000
PM-B01	Weihai	Feces	ST300	JCBAFG000000000
PM-B02	Weihai	Feces	ST204	JCBAFF000000000
PM-B03	Weihai	Feces	ST178	JCBAFE000000000
PM-B04	Weihai	Feces	ST135	JCBAFD000000000
PM-B05	Weihai	Feces	ST421	JCBAFC000000000
PM-B06	Weihai	Feces	Unassigned*	JCBAFB000000000
PM-B09	Weihai	Feces	ST266	JCBAFA000000000
PM-B12	Weihai	Feces	ST135	JCBAEZ000000000
PM-B13	Weihai	Feces	ST204	JCBAEY000000000
PM-B14	Weihai	Feces	ST135	JCBAEX000000000
PM-B15	Weihai	Feces	ST135	JCBAEW000000000
PM-C03	Weihai	Intestinal contents	ST135	JCBAEV000000000
PM-C06	Weihai	Intestinal contents	ST336	JCBAEU000000000
PM-C07	Weihai	Intestinal contents	ST185	JCBAET000000000
PM-C13	Weifang	Intestinal contents	ST245	JCBAES000000000
PM-C14	Weifang	Intestinal contents	ST178	JCBAER000000000
PM-C20	Weifang	Intestinal contents	ST212	JCBAEQ000000000
PM-C22	Weifang	Intestinal contents	ST135	JCBAEP000000000
PM-C26	Weifang	Intestinal contents	ST185	JCBAEO000000000
PM-C28	Weifang	Intestinal contents	ST135	JCBAEN000000000
PM-C37	Weifang	Intestinal contents	ST188	JCBAEM000000000
PM-C42	Weifang	Intestinal contents	ST185	JCBAEL000000000

Isolate ID	Sampling location	Sample type	Sequence type (ST)	GenBank WGS accession
PM-C48	Weifang	Intestinal contents	ST336	JCBAEK000000000
PM-C49	Weifang	Intestinal contents	ST135	JCBAEJ000000000
PM-C65	Weifang	Intestinal contents	ST185	JCBAEI000000000
PM-C68	Weifang	Intestinal contents	ST336	JCBAEH000000000
PM-C70	Weifang	Intestinal contents	ST423	JCBAEG000000000
PM-C72	Weifang	Intestinal contents	ST423	JCBAEF000000000
PM-C74	Weifang	Intestinal contents	ST210	JCBAEE000000000
PM-C79	Weifang	Intestinal contents	ST210	JCBAED000000000
PM-C80	Linyi	Intestinal contents	ST423	JCBAEC000000000
PM-C109	Linyi	Intestinal contents	ST35	JCBAEB000000000
PM-F06	Yantai	Feces	ST85	JCBAEA000000000
PM-F09	Yantai	Feces	ST523	JCBADZ000000000
PM-F11	Yantai	Feces	ST188	JCBADY000000000

Note: All draft genome assemblies were deposited under NCBI BioProject accession PRJNA1494192.
*For PM-B06, the partial MLST profile was *atpD*(−), *dnaJ*(135?),*mdh*(172?), *pyrC*(79?), *recA*(105?), and *rpoD*(−); therefore, no ST was assigned. "?" indicates a partial match to the indicated PubMLST allele, whereas "−" indicates that no callable allele was recovered from the draft assembly.

Supplementary Table S4. Minimum inhibitory concentration (MIC) interpretive criteria and antimicrobial-category assignments used for antimicrobial susceptibility testing and MDR classification of mink-derived *Proteus mirabilis* isolates.

Antimicrobial agent	Susceptible MIC (μg/mL)	Intermediate / SDD MIC (μg/mL)	Resistant MIC (μg/mL)	Interpretive source	Antimicrobial category for MDR classification
Doxycycline (DO)	≤4	8	≥16	A	Tetracyclines (excluded)
Imipenem (IPM)	≤1	2	≥4	A	Carbapenems
Cefepime (FEP)	≤2	4–8 (SDD)	≥16	A	Extended-spectrum cephalosporins
Gentamicin (GEN)	≤4	8	≥16	A	Aminoglycosides
Amikacin (AMK)	≤16	32	≥64	A	Aminoglycosides
Enrofloxacin (ENR)	≤0.5	1	≥2	B	Fluoroquinolones
Cefoxitin (FOX)	≤8	16	≥32	A	Cephamycins
Ampicillin (AM)	≤8	16	≥32	A	Penicillins
Florfenicol (FFC)	≤4	8	≥16	C	Phenicols
Trimethoprim–sulfamethoxazole (SXT)	≤2/38	Not defined	≥4/76	A	Folate pathway inhibitors
Aztreonam (ATM)	≤4	8	≥16	A	Monobactams

Note: S, susceptible; I, intermediate; R, resistant; SDD, susceptible-dose dependent.
A: MIC interpretive criteria for Enterobacteriaceae presented in CLSI VET08, 4th ed. (2018), Table 2A, and adopted from CLSI M100, 28th ed. (2018)[14,15].
B: Because no mink-specific breakpoint was available, the FDA-approved canine/feline enrofloxacin criterion was applied as a surrogate for phenotypic categorization[16].
C: Because no mink-specific breakpoint was available, the swine-derived florfenicol MIC criterion for Enterobacterales was applied as a surrogate for epidemiological interpretation only[17].
D: Antimicrobial-category assignments followed an adaptation of the framework proposed by Magiorakos et al. [18]. ENR and FFC were included in the MDR calculation as fluoroquinolone and phenicol agents, respectively. DO was excluded because intrinsic resistance to tetracyclines was not considered acquired resistance.

Supplementary Table S5. Average nucleotide identity (ANI) analysis of the 39 sequenced mink-derived *Proteus mirabilis* isolates.

Isolate ID	ANI (%)
PM-A28	98.93
PM-A32	98.96
PM-A35	99.08
PM-A36	99.11
PM-B01	98.74
PM-B02	99.11
PM-B03	98.88
PM-B04	99.11
PM-B05	99.11
PM-B06	98.44
PM-B09	99.07
PM-B12	99.15
PM-B13	99.13
PM-B14	99.04
PM-B15	99.01
PM-C03	99.05
PM-C06	98.99
PM-C07	99.19
PM-C13	98.86
PM-C14	99.08
PM-C20	98.83
PM-C22	99.17
PM-C26	99.25
PM-C28	99.11
PM-C37	99.16
PM-C42	99.24
PM-C48	99.00
PM-C49	99.01
PM-C65	99.16
PM-C68	99.01
PM-C70	99.11

Isolate ID	ANI (%)
PM-C72	99.13
PM-C74	99.05
PM-C79	98.94
PM-C80	99.11
PM-C109	98.97
PM-F06	99.18
PM-F09	99.14
PM-F11	99.11

Note: ANI, average nucleotide identity. Values were calculated using skani v0.3.2 in slow mode against the genome assembly of the *P. mirabilis* type strain ATCC 29906 (GCF_000160755.1). An ANI value of ≥95% was used as the species-level threshold.
